# Will colleges survive the storm of declining enrollments? A computational model

**DOI:** 10.1371/journal.pone.0236872

**Published:** 2020-08-10

**Authors:** Oleg V. Pavlov, Evangelos Katsamakas

**Affiliations:** 1 Department of Social Science and Policy Studies, Worcester Polytechnic Institute, Worcester, MA, United States of America; 2 Gabelli School of Business, Fordham University, New York, NY, United States of America; Universidad de los Andes, COLOMBIA

## Abstract

The approaching decline in the U.S. college-age population, sometimes referred to as a “*demographic storm*,” has been identified as an existential threat to the future of American colleges and universities. This article conducts a *model-driven analysis* of three plausible college-level responses to declining applications. It draws on *systems theory* to conceptualize a tuition-dependent college as a complex service system and to develop a system dynamics model that captures key causal interrelationships and multiple feedback effects between faculty, facilities, tuition revenue, financials, reputation, and outcomes. Simulations with the *college model* suggest that common solutions such as reducing faculty or adding campus facilities may improve the college’s short-term financial position, but they are insufficient to ensure its long-term viability. This model contributes to the research literature on the economics of higher education, and model-driven academic management and strategy. It also provides useful implications and insights that can inform policy-makers and college leaders.

## Introduction

There are nearly four thousand degree-granting institutions in the United States [1]. They range from highly selective global research universities with tens of thousands of students to small community colleges with open admission. These institutions of higher education face many challenges. One existential threat is the approaching decline in the U.S. college-age population, sometimes referred to as a “demographic storm” [2]. The prospects are especially dire for tuition-dependent private colleges [3, 4], and some observers have predicted that half of American colleges and universities will soon perish [5]. In this context, college leaders seek to understand how to adapt to declining student applications [4, 6–9]. State governments and policy-makers would also benefit from insight into this problem to prevent escalating college closures [10] and the associated negative impact on the U.S. economy.

We describe the problem of declining enrollments and frame it using a computational model. The article approaches colleges as complex systems that provide educational services [11–16]. The system view of academic institutions studies the dynamics of education provision by understanding how the elements of the institution interact in response to changes in the operating environment. The theory developed here is implemented as a *system dynamics computational model* [17, 18] that includes causal feedback mechanisms between students, faculty, facilities, and college financials. Thus, the article contributes to the literature on the economics of higher education [2, 4, 12], and *model-driven academic management and strategy* [19–21].

The proposed model allows us to conduct a *model-driven analysis* of three plausible college-level responses to declining applications. The “do-nothing” scenario serves as the base case. The remaining two scenarios investigate strategies aimed at cutting costs and increasing revenue. A common cost-cutting strategy is to reduce the number of teaching faculty [3, 7, 22, 23]. The third scenario examines a revenue increase strategy, according to which a college attempts to attract more students by offering better facilities [3, 6, 7, 22, 24]. Overall, this analysis provides useful implications and insights that can inform policy-makers and college leaders.

The next section describes the problem of declining student applications and how colleges have tried to overcome it. Then, we review the study method. Next, we build a system dynamics model of a representative tuition-dependent college. Lastly, we use the computational *college model* to analyze three scenarios, discuss results and insights, and propose extensions for future research.

## The problem of declining enrollments

Nationwide trends indicate that the college-age population in the U.S. will drop between 13 and 29 percent depending on the state in the next ten years [2, 8]. For example, in Massachusetts, the number of high school graduates is projected to drop by about 15 percent within a decade [7]. The demographic decline is likely to translate into lower enrollments and operating deficits at tuition-dependent colleges [4]. Moreover, operating expenditures per-student will increase because the costs of running a college will spread across fewer students [4]. Declining enrollments is terrible news for many private colleges that are often teetering on the brink of closure [22]. Historically, institutions attempted to resolve operational deficits by increasing revenue and cutting expenses, as the following examples demonstrate.

Looking back to the 1990s, Townsley [22] recounts stories of several colleges that struggled with declining enrolments and failed. For example, Bradford College in Massachusetts tried maintaining enrollments through the 1980s and 1990s by adding new majors and offering generous financial aid. Before permanently closing, it offered 40 majors while having only 35 faculty members. Between 1988 and 1998, the share of the revenue from tuition and fees given back as financial aid, called the discount rate, increased from 19 to 48 percent. Operating deficits continued through the 1990s. In 1999, the deficit was $6.1 million on an annual budget of $14 million. In 1998, the college took an $18 million loan to refinance old debt and to build a new dormitory hoping that the new building would attract students and increase enrollment. However, enrollments did not improve, and the college closed in 2000.

Another failed college reviewed by Townsley [22] is Trinity College, a women’s college, which operated in Vermont. From 1990 to 1999, enrollment in continuing education and undergraduate programs dropped by about 30 percent, despite a discount rate as high as 45 percent. When the operating deficit reached $2.7 million, the college cut 20 of 30 majors and kept only ten faculty members. In academic year 1999–2000, about 60 students enrolled, which forced the college to close in 2000.

More recently, Rivard [23] provides an account of 15 small private colleges that responded to financial troubles due to lackluster student recruitment with dramatic cuts in faculty and staff accompanied by program closures. For example, Midway College in Kentucky laid off about 30 percent of its 54 faculty. Holy Family University in Philadelphia let go of 20 percent of its 100 faculty members in addition to cutting staff positions. Similarly, Anderson University in Indiana reduced its faculty by four percent. Wittenberg University in Ohio reduced faculty positions by 21 percent.

**Fig 1:**
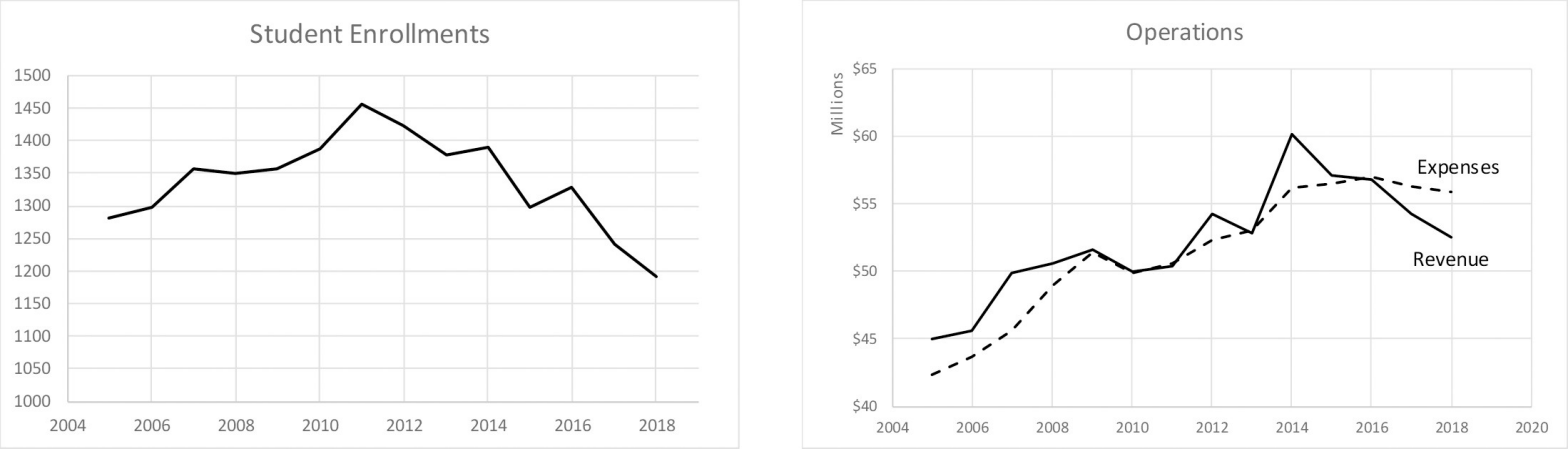
Student enrollments, revenue, and operating expenses at Hampshire College. Data sources: Financial reports of Hampshire College for 2005–2018 (available at https://www.hampshire.edu/business-office/financial-reports; Last accessed on April 15, 2020), [28, 29].

Hampshire College in Amherst, Massachusetts, provides a striking recent example of the challenges that tuition-dependent colleges face. It admitted its first students in 1970. The college is known as an experiment in self-directed education because it has no grades, majors, or traditional departments [25–27]. Nearly 90 percent of the revenue comes from tuition and fees [28]. As student enrollments declined by 20 percent between 2014 and 2019, revenue dropped from $60 million to $49 million (Fig 1). Due to decreasing enrollments, the college has been experiencing operating deficits since 2016. The college used major gifts and emergency endowment withdrawals to address these deficits. Moreover, it responded by reducing faculty and staff and by cutting operating expenses [28]. It still expects a deficit of $20 million by 2022, which might lead to a closure or a merger with another institution.

## Method

Higher education management and resource planning is a complex task that involves balancing the wishes of multiple stakeholders [12, 14, 22]. Despite its complexity, academic planning is still often performed with minimal analytical backing. Model-driven academic planning is an improvement over traditional methods because it allows academic stakeholders to consider alternatives and review the dynamics of plausible scenarios before making a decision [19]. The first models for academic planning were developed in the 1960s [30, 31]. Early planning tools relied on spreadsheet models [32]. Eliman [33] combined a statistical regression model and a linear programming model to estimate the supply of school graduates, demand for university spots, and determine the allocation of students. Strategic planning tools also used the Markov chain models to simulate student performance [34] and economic input-output models for resource planning on campus [35].

Researchers have been advocating for using the systems approach for academic planning because it is well-suited for modeling the complex and dynamic nature of higher education [12, 13, 20, 36–38]. Therefore, this article adopts system dynamics to model operations of a college. System dynamics is a modeling methodology that recognizes circular chains of causality that form feedback loops and introduce delays [17, 39]. Besides quantitative variables, system dynamics models can include qualitative measures, such as the reputation of an institution, which are important in higher education [20]. Since its introduction in the late 1950s [40], system dynamics has become the second most widely used modeling approach in operations research [41] with applications in many fields, including management (e.g. [18, 42]), strategy (e.g. [17, 43]), political science (e.g. [44–46]) and health (e.g. [47, 48]). Many universities worldwide offer courses and academic programs in system dynamics [49–52].

The system dynamics approach [17, 18, 53] involves building a computational model in several iterative steps. First, the problem is clearly stated, which means that the simulated time range and behaviors that need to be examined are identified. The time range and the behaviors determine the level of analysis and the model boundary. During the second step, the modeler lists variables to be included in the model. For example, this article performs analysis at the college level, and therefore environmental factors that are beyond college control are assumed as external to the model. Third, based on the research literature, field work or interviews with domain experts, causal relationships between variables are documented using the pictorial notation [54] similar to the notation used for signed directed graphs. In the fourth step, the causal structure developed in the previous step is implemented as a computational model. System dynamics models are usually built and simulated in specialized modeling software such as *Stella Architect* (sold by *isee systems*), *Vensim* (offered by *Ventana Systems*), or *Powersim* (sold by *Powersim Software*). This study uses *Stella Architect*. Mathematically, a system dynamics model is a set of nonlinear, non-stochastic integral equations that are solved numerically by the modeling software. The computational model is used to simulate scenarios that test public policies or management strategies.

System dynamics has been used for high-level policy planning as well as studies at the college level [55]. For example, Galbraith [21] analyzed the effects of national educational policies in Australia. Strauss and Borenstein [56] built a system dynamics model to explore difficulties in achieving national educational goals in Brazil. Bergland et al. [57] forewarned the administration of a college of an upcoming budgetary collapse due to the student admission policies. Zaini et al. [58] modeled the strategic resource allocation at a new university in Russia. Barlas and Diker [59] used an interactive system dynamics model to analyze long-term management of enrollment, number of faculty, teaching quality, research output, and outside consulting. Oyo et al. [60] studied the impact of government funding schemas on university capacity and productivity in a developing country. Sahay and Kumar [61] used system dynamics to investigate “what-if” scenarios for teaching quality improvement at a university.

This article adapts and extends an earlier model [62], which was built, validated and calibrated in consultations with key stakeholders at a private university. We generalize the previous model in order to study the financial viability of tuition-dependent colleges. We add financial details, which are informed by the relevant literature on college economics and management. The following section develops the *college model* that describes the operation of a typical tuition-based college.

### The college model

The *college model* consists of four interconnected sectors: Students, Faculty, Facilities, and Financials. This section describes the causal structure of each sector, while the mathematical equations and parameter values are in the Appendix B.

#### Students

**Fig 2:**
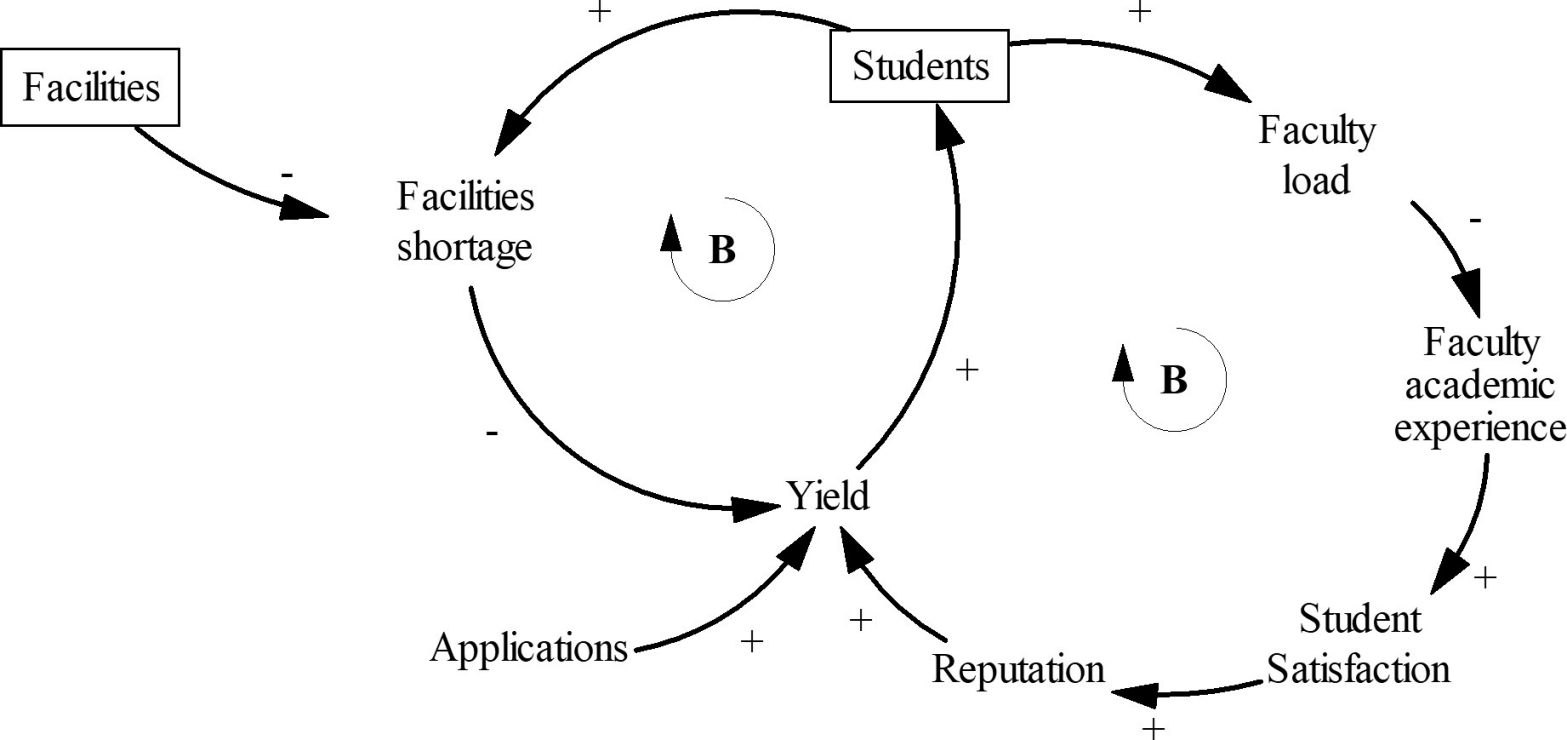
Causal structure of the *Students* sector. Rectangles indicate stocks. Arrows show direction and polarity of causal relationships. The letter B signifies balancing loops.

The students sector (Fig 2) represents student enrollments and several factors that affect it. Arrows indicate causal directionality [54]. The arrow is positive when the cause and effect variables change in the same direction. When the cause and effect move in the opposite directions, the link is negative. Rectangles indicate variables that accumulate, called stocks. Stocks describe the state of the system. Mathematically, stocks are integral equations, which introduce inertia and delays in the system. Circular causal connections form feedback loops. The letter B indicates a balancing (negative) feedback loop, while the letter R indicates a reinforcing (positive) loop. Fig 2 shows two balancing loops. Balancing loops add stability to the system.

The model assumes that there is an exogenous number of applications every year. Of the admitted applicants, only a fraction, called yield, eventually enrolls at the college [63]. The new students join the existing stock of students. The model includes two factors that affect the enrollment decision: academic reputation of the college and the adequacy of campus facilities expressed as facilities shortage. Facilities shortage is reduced when the stock of facilities increases. Colleges compete for students by investing in facilities [6, 7, 22].

Academic reputation depends on the faculty [4]. Assuming that this is an undergraduate college, we exclude research. The number of faculty and students determine the faculty teaching load, which increases as more students arrive on campus and decreases as the college hires more faculty. High faculty teaching loads lower the academic experience of faculty, student satisfaction, and the college reputation.

#### Faculty

**Fig 3:**
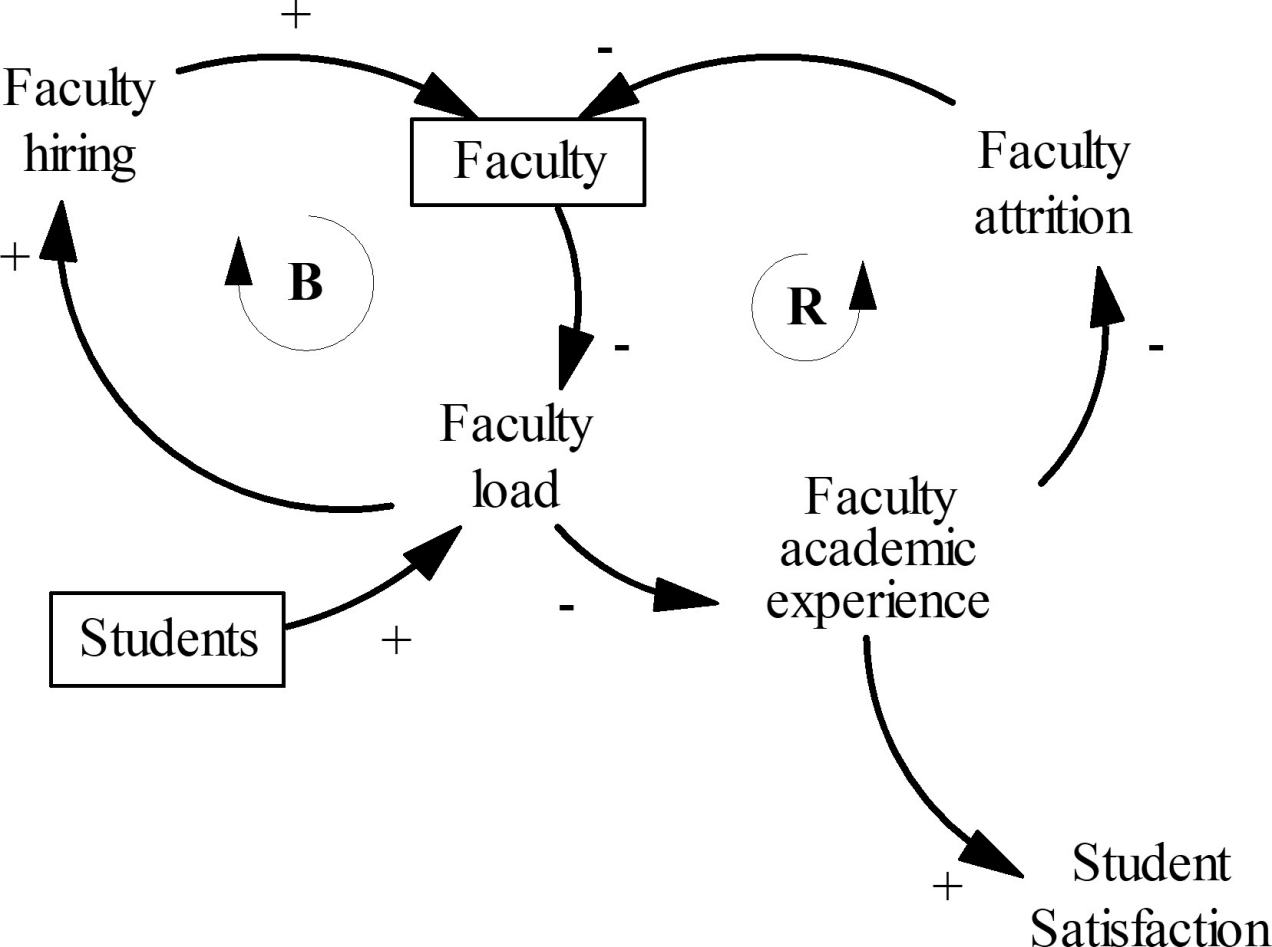
Causal structure of the faculty sector. The letter B signifies a balancing loop. The letter R is for a reinforcing loop.

The faculty teaching load increases when there are more students (Fig 3). If the college does not address the high faculty load issue, then the faculty academic experience deteriorates, which affects morale leading to faculty attrition. As professors leave the university, the stock of faculty decreases, and therefore the teaching load of the remaining faculty increases even further, which again degrades the academic experience of the faculty. This circular causation forms a reinforcing loop marked by the letter R, a vicious cycle, which can drag down the academic experience on campus. To lower the teaching load, the college can hire more professors–this is the balancing loop in Fig 3.

#### Facilities

**Fig 4:**
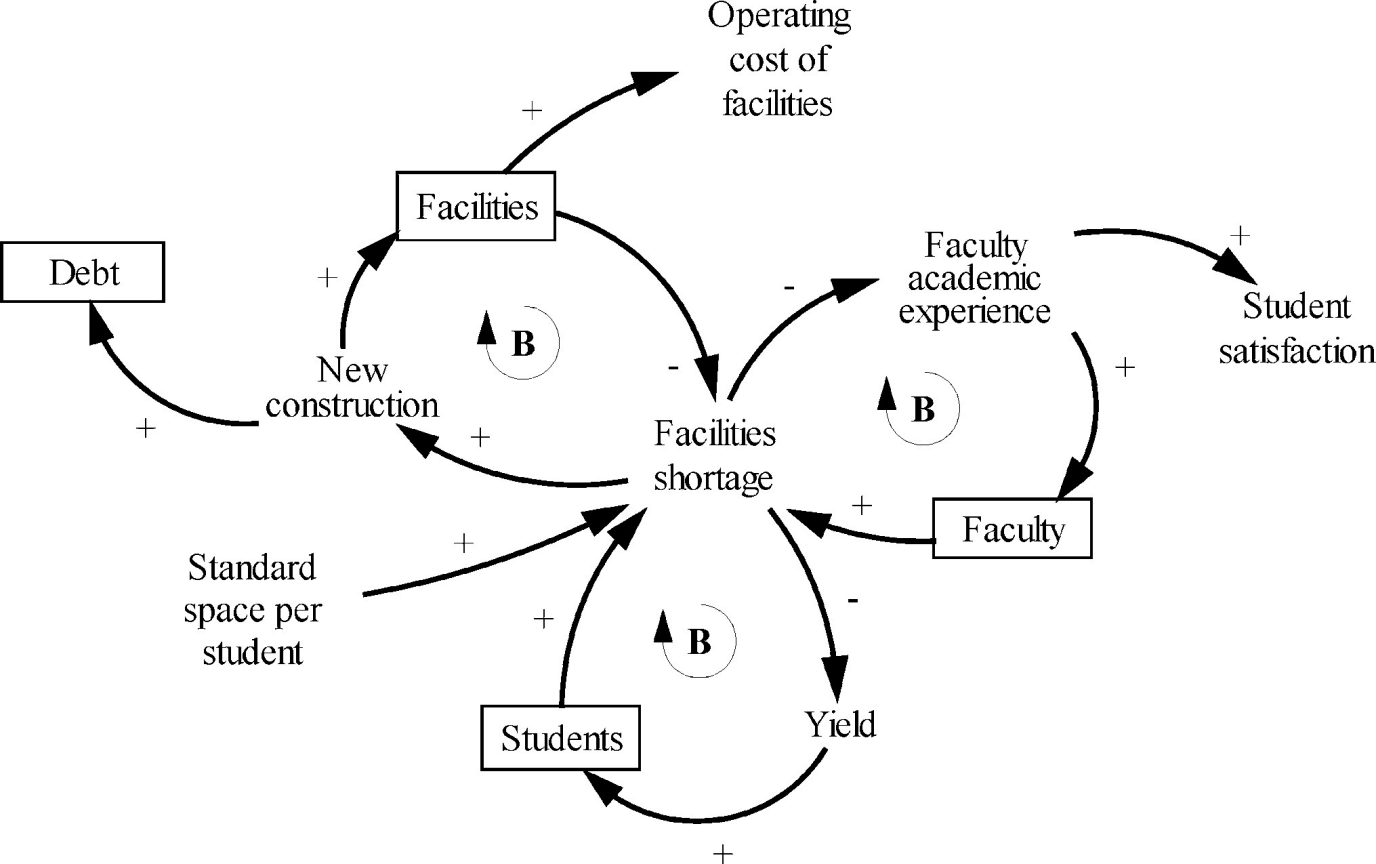
Causal structure of the facilities sector.

Facilities planning is one of the primary strategic responsibilities of academic leadership [24]. Facilities include dorm rooms for students, classrooms, and laboratories for teaching, and office space for faculty. More faculty and students may lead to a facilities shortage, a problem that the college can address through new construction (Fig 4). However, because capital projects are complex undertakings that involve many stakeholders and take many years of planning, fundraising, and construction, available space often lags behind the desired space, especially in times of growing or declining enrollment [22, 24].

Most of the capital funding comes in the form of loans [24]. Therefore, this model assumes that the college constructs facilities with borrowed funds. Maintenance and operation of the facilities add to the operating cost. Facilities shortage negatively impacts faculty academic experience.

#### Financials

**Fig 5:**
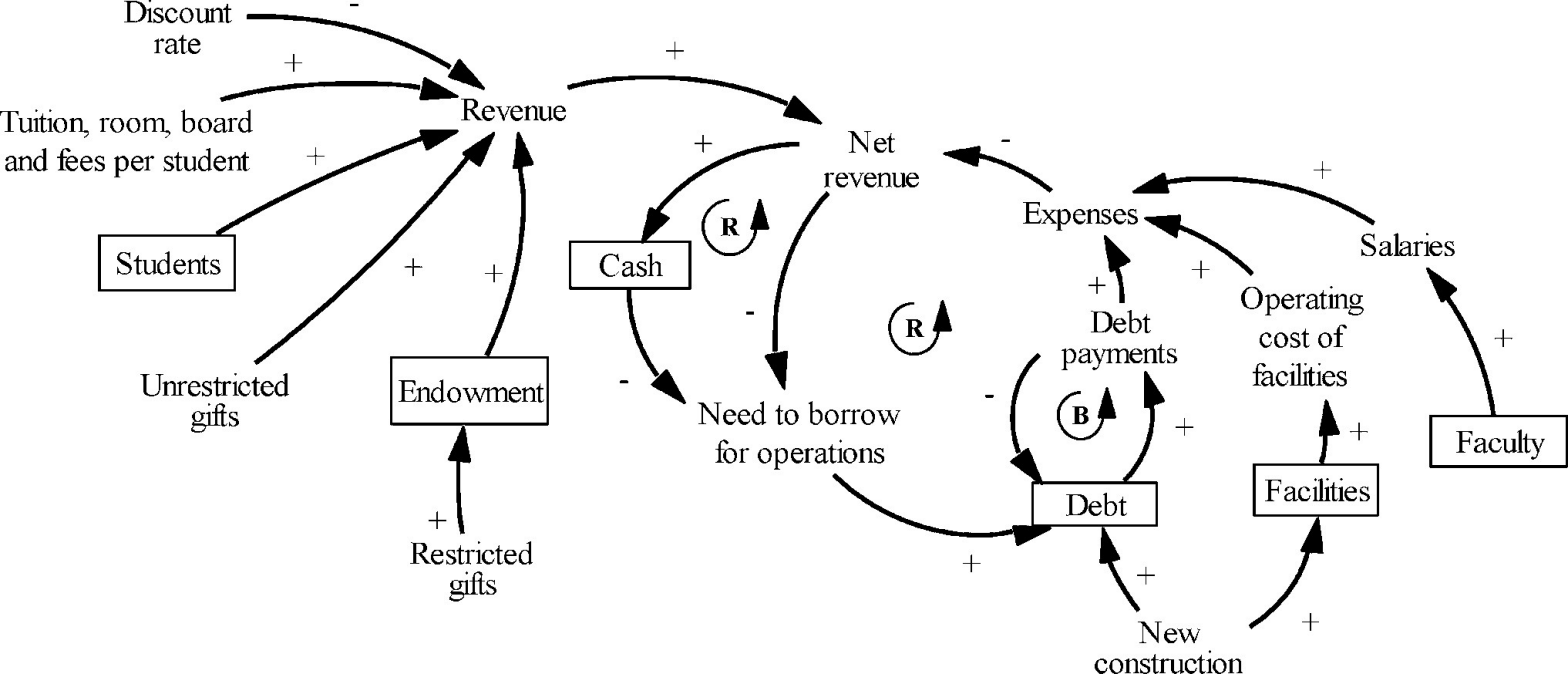
Causal structure of the financial sector.

While college finances are complex and intertwined [12, 22, 64], for simplicity, this model includes only three financial stocks: the emergency reserve of cash, endowment, and debt (Fig 5). Tuition, room, board, and fees are the main contributors to the revenue and are assumed to be constant. Future versions of the model can relax this assumption. The discount rate is the fraction of tuition, room, board, and fees given back to some students in the form of financial aid. Providing financial aid reduces the amount of money that the college has for operational expenses [4, 22]. Some unrestricted gifts can be used for operations.

Expenses include faculty salaries, cost of operating facilities, and debt payments. The difference between revenue and expenses constitutes the net revenue. Maintaining a cash reserve for a “rainy day” is one of the approaches to strengthening the financial health of a college [3]. The model assumes that the college maintains a stock of cash, which is replenished when the college has an operating surplus and depleted when the college needs to use the cash for operations. When the college borrows for operations or new construction, new loans add to the stock of the existing debt.

**Fig 6:**
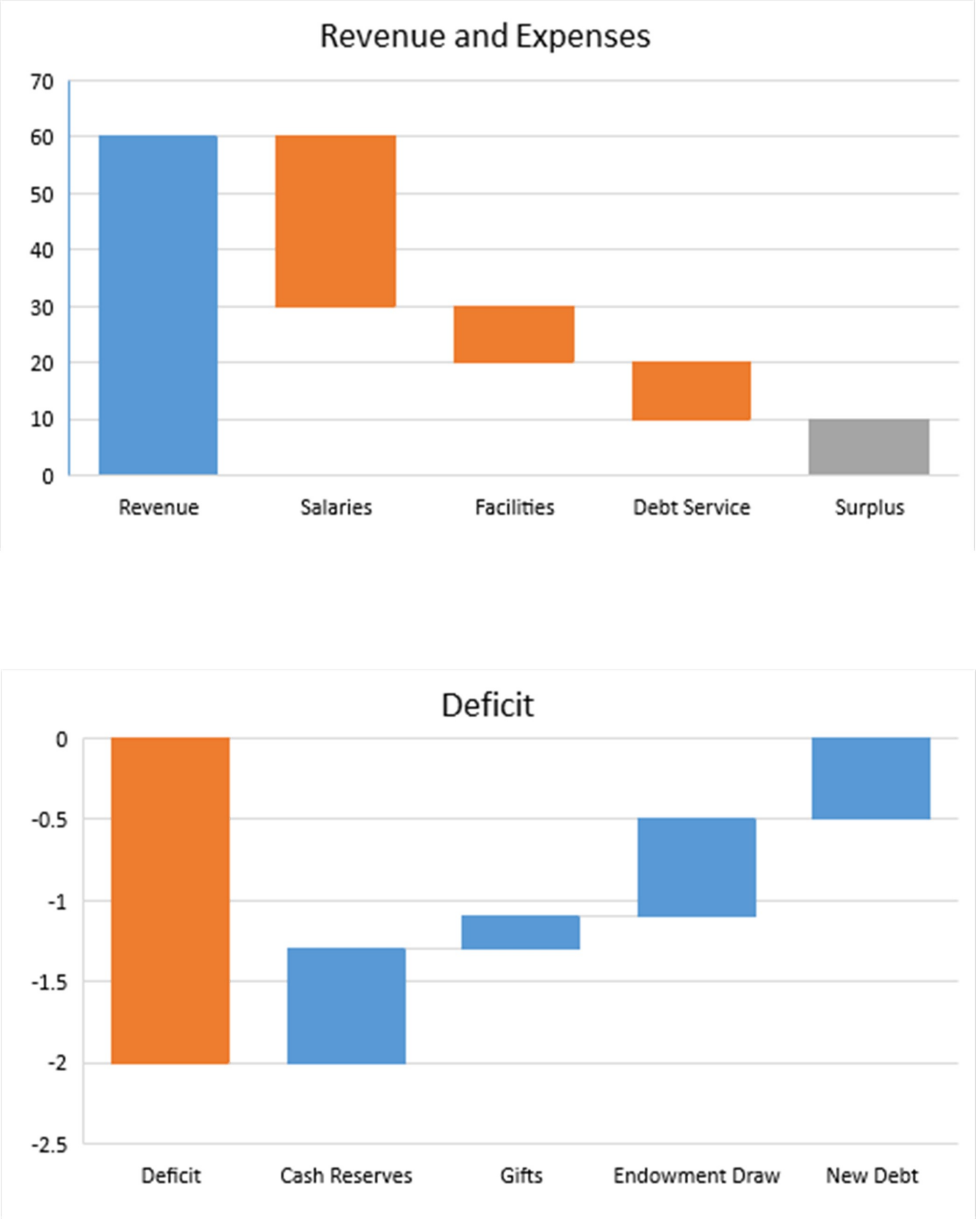
Assumptions about components of the net revenue. The graphs show two cases: (a) the revenue is $60 million, expenses are $50 million, and the surplus is $10 million; and (b) the operating deficit of $2 million is covered from the cash reserve, gifts, endowment draws and borrowing.

In the example in Fig 6A, the revenue of $60 million is spent on salaries, operating facilities, and paying off debt; the remainder is the surplus. If operating expenses are higher than the revenue, then the net revenue is negative, and it is called the operating deficit. It can be covered by drawing from the cash reserve, unrestricted gifts, endowment withdrawals, and borrowing for operations (Fig 6B). We assume that the internal university rules limit the percentage of the endowment that can be withdrawn every year. The endowment can be increased with new gifts.

#### Complete model

**Fig 7:**
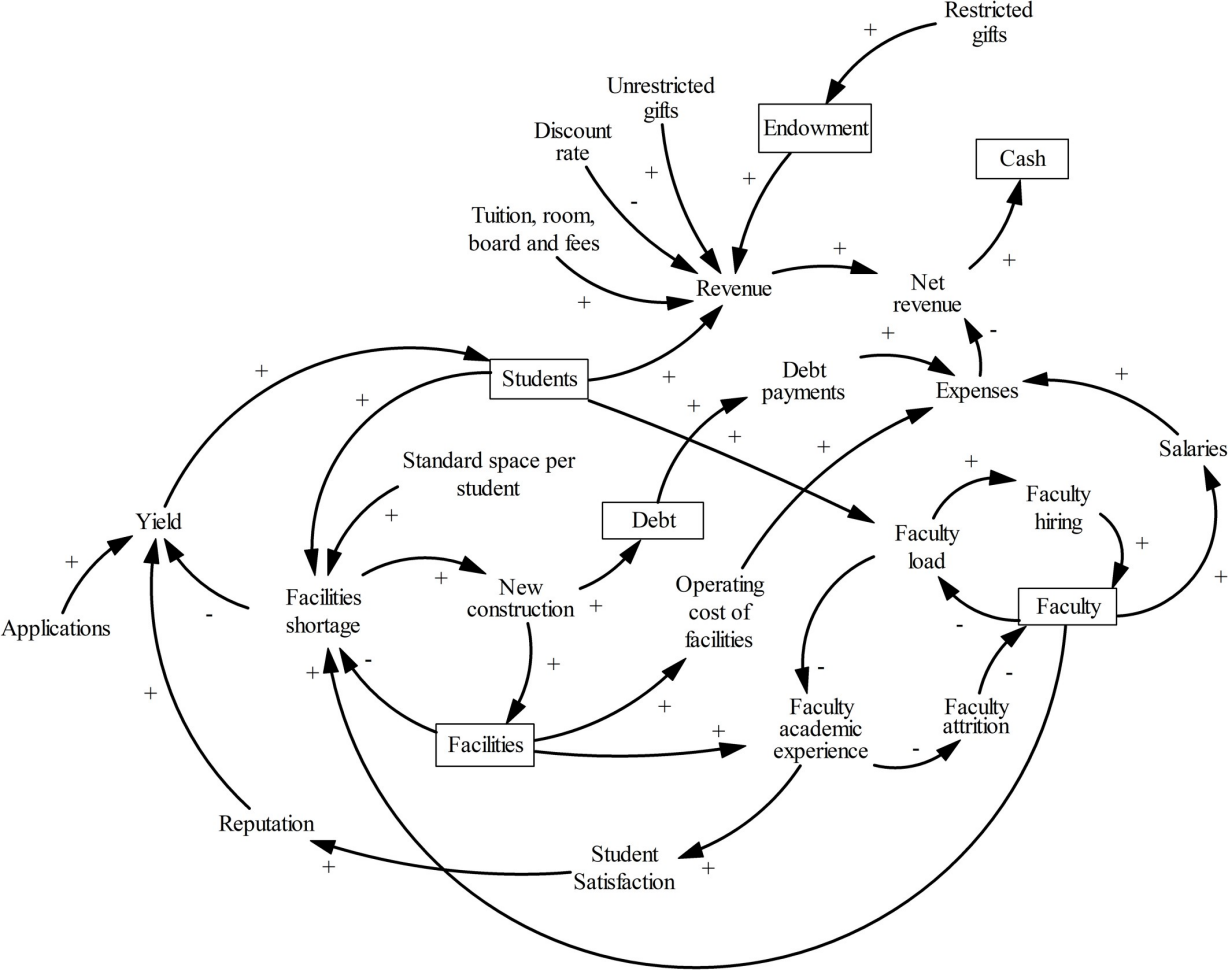
Causal structure of the *college model*. The model has six stocks, shown as rectangles.

Fig 7 shows the causal structure of the entire *college model*, which consists of the four sectors detailed above. This model captures the many causal and feedback effects between elements of a representative tuition-based college, which include faculty, facilities, tuition revenue, endowment, debt, reputation, and educational outcomes. The system model tracks numerous simultaneous effects triggered by the external operating environment and by the management decisions.

### Scenario analysis

This section uses the *college model* to study three possible responses to declining student applications and operating deficits. The first simulated scenario examines the “do nothing” strategy, which serves as the base case. The second strategy aims to lower operating costs by reducing the number of faculty. The goal of the third strategy is to increase revenue by attracting more students when the college improves its facilities. These cost and revenue strategies are popular with stressed colleges, as has been discussed earlier in this article. Colleges may pursue additional strategies, but they are not considered in this article and will be studied in future research.

**Fig 8:**
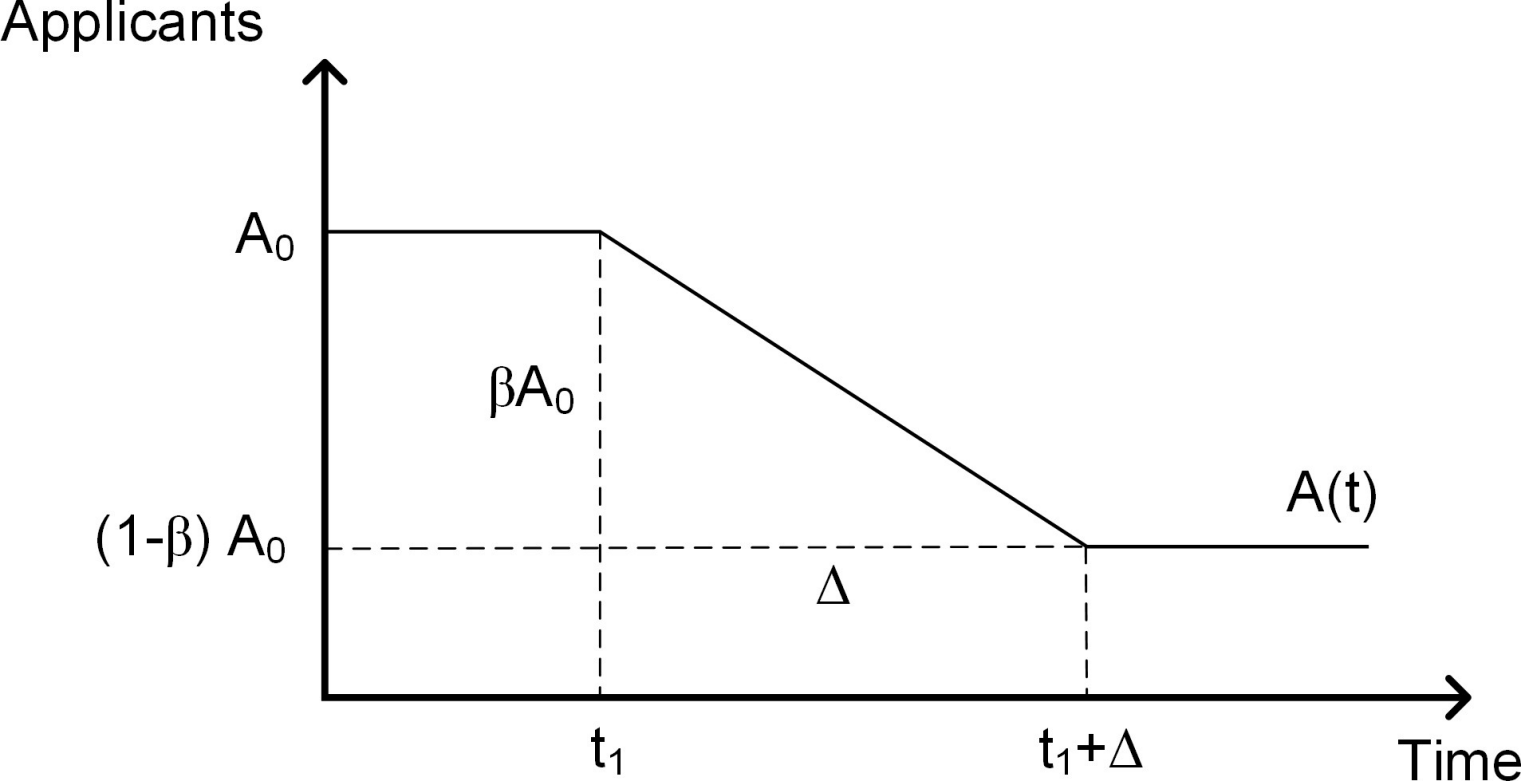
Drop in applications.

We model the demographic decline as an external variable using the function in Fig 8. Here, we consider a 15 percent application drop, which is the situation predicted for Massachusetts [7]. Massachusetts has recently seen a slew of college closures, which warranted serious concerns at the regulatory level [10]. Appendix A shows simulation results for different decline rates. All simulations start in equilibrium in 2010 when the college receives *A*_0_ = 6,500 applications per year. Simulations run for 15 periods through 2025. We assume that applications start to decline in year *t*_1_ = 2015 and they decline over the following ten years, that is Δ = 10 years. Since this section assumes a decline rate of 15 percent, i.e. β = 0.15, after 10 years applications drop to (1−*β*)*A*_0_ = 0.85*6,500 = 5,525 applications per year. To isolate the effects of the three strategies, we assume away gifts, interest on debt, and market returns on endowment. In all scenario simulations, we introduce interventions in 2015, *ceteris paribus*. We compare the short-term (two years) and long-term (10 years) outcomes.

#### Scenario 1: Do nothing

**Fig 9:**
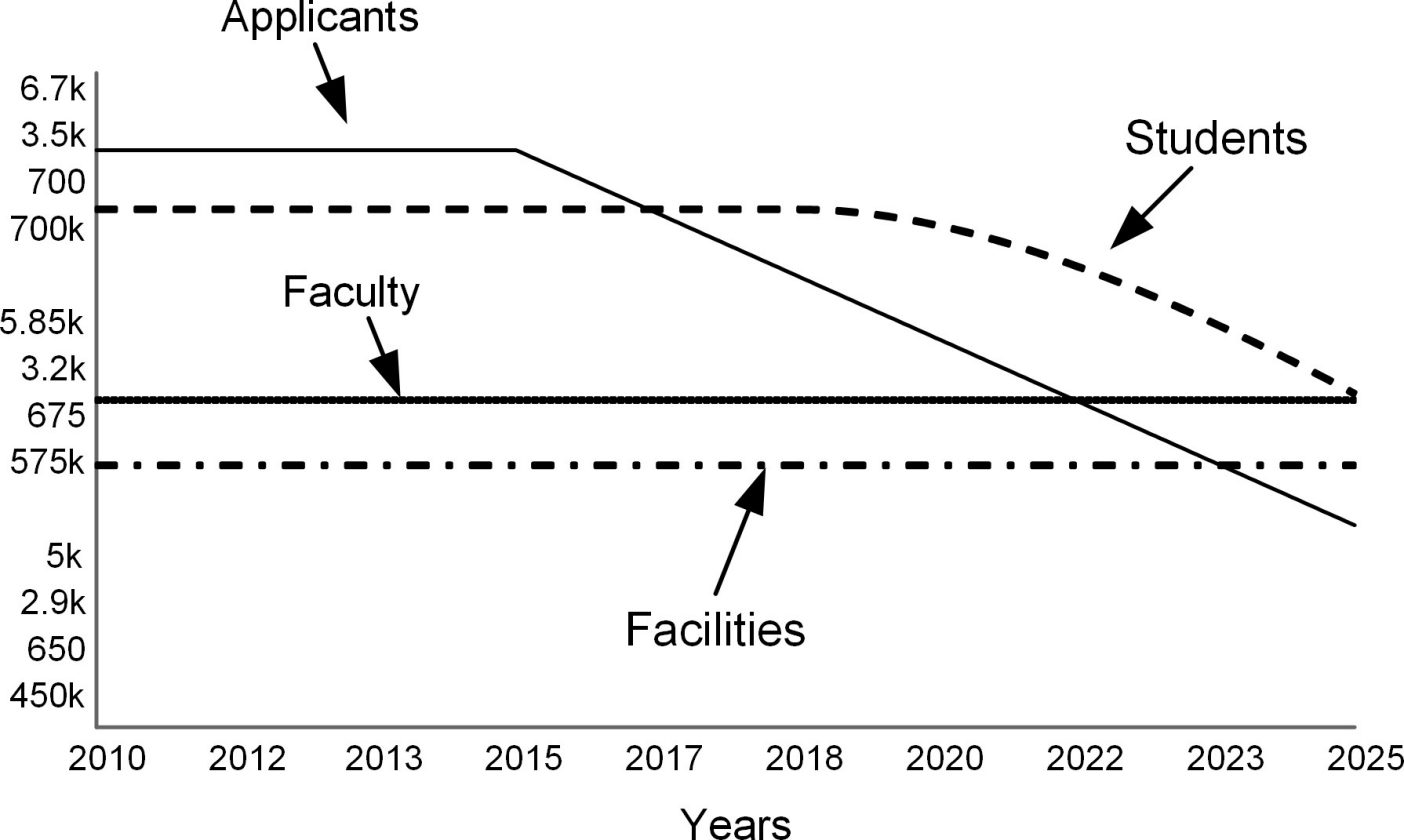
As applications drop by 15% over ten years, student enrollments decline. Faculty and facilities stay unchanged.

The first run simulates the situation when the college does not actively mitigate the declining applications. The “do nothing” scenario demonstrates the adverse effects of the demographic decline. As applications drop (solid line in Fig 9), so does the number of enrolled students (dashed curve in Fig 9). In this scenario, the stocks of faculty and facilities remain constant.

**Fig 10:**
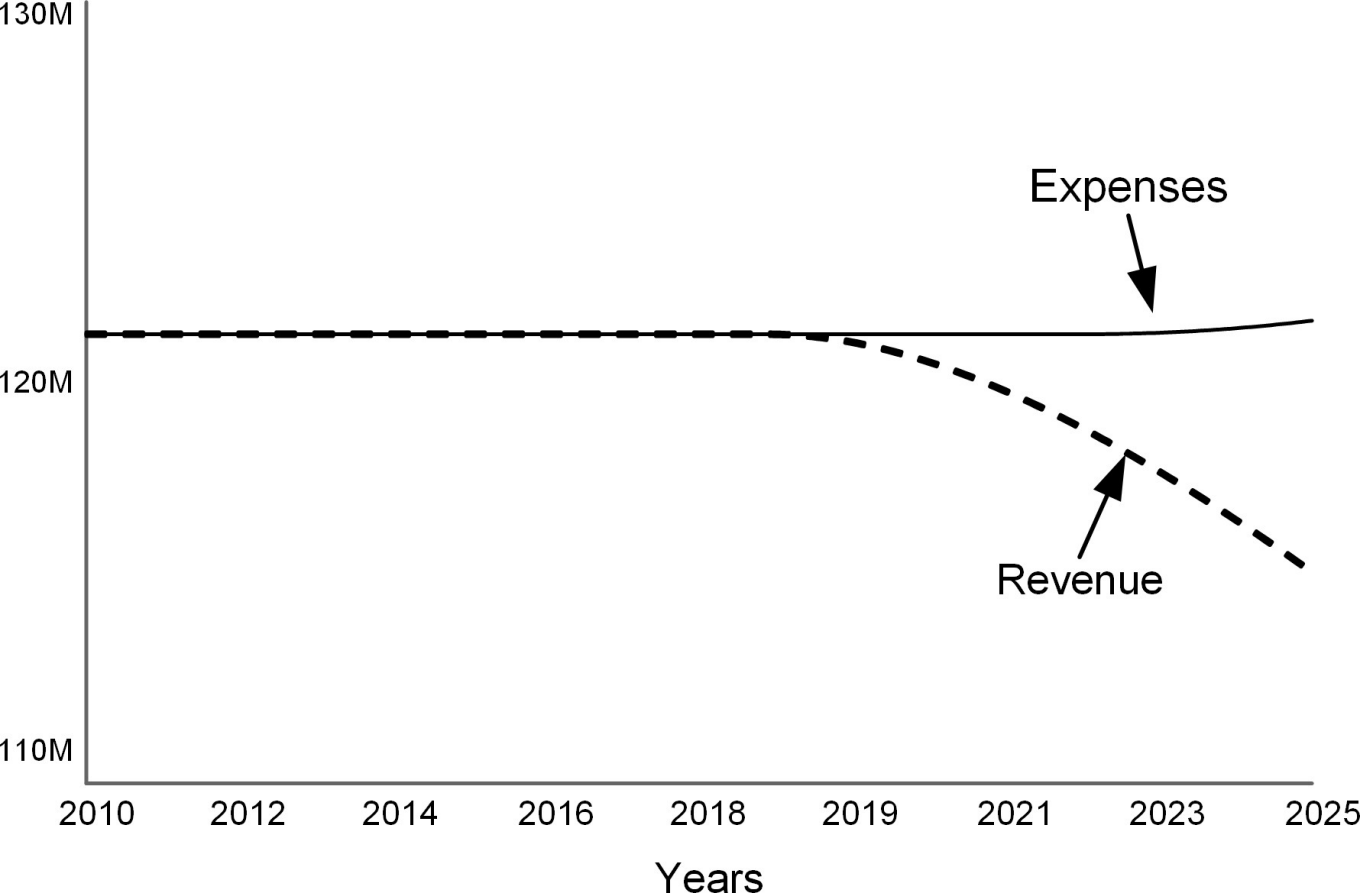
Revenue and expenses.

**Fig 11:**
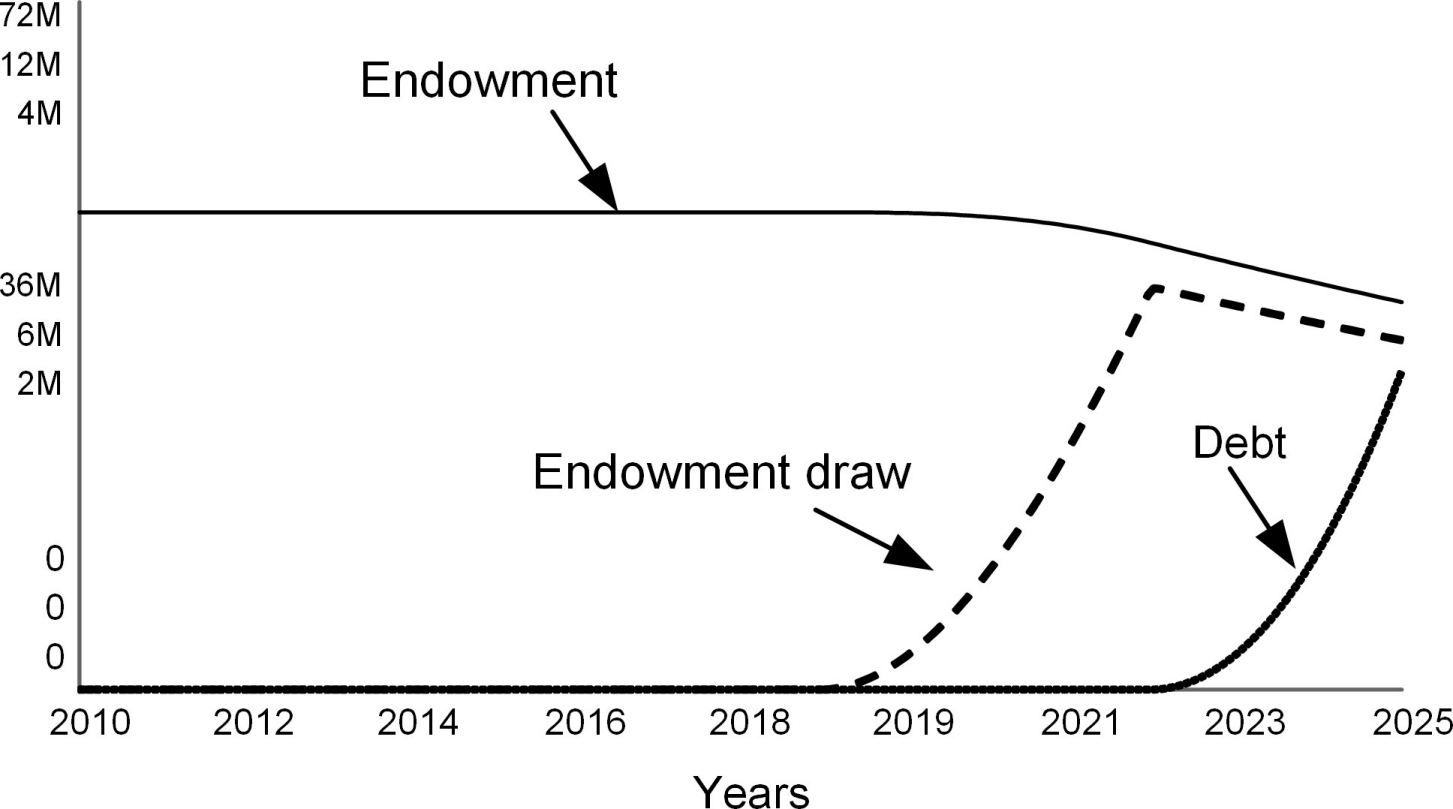
Endowment and debt.

Lower student enrollments hurt the revenue (dashed curve in Fig 10) and cause yearly deficits after 2018 (see the gap between revenue and expenses in Fig 10). The college resolves annual deficits by withdrawing from the endowment and, when the endowment draw is not sufficient beyond 2022, by borrowing (see Fig 11). A bigger debt requires higher interest payments that add to the operating expenses.

**Fig 12:**
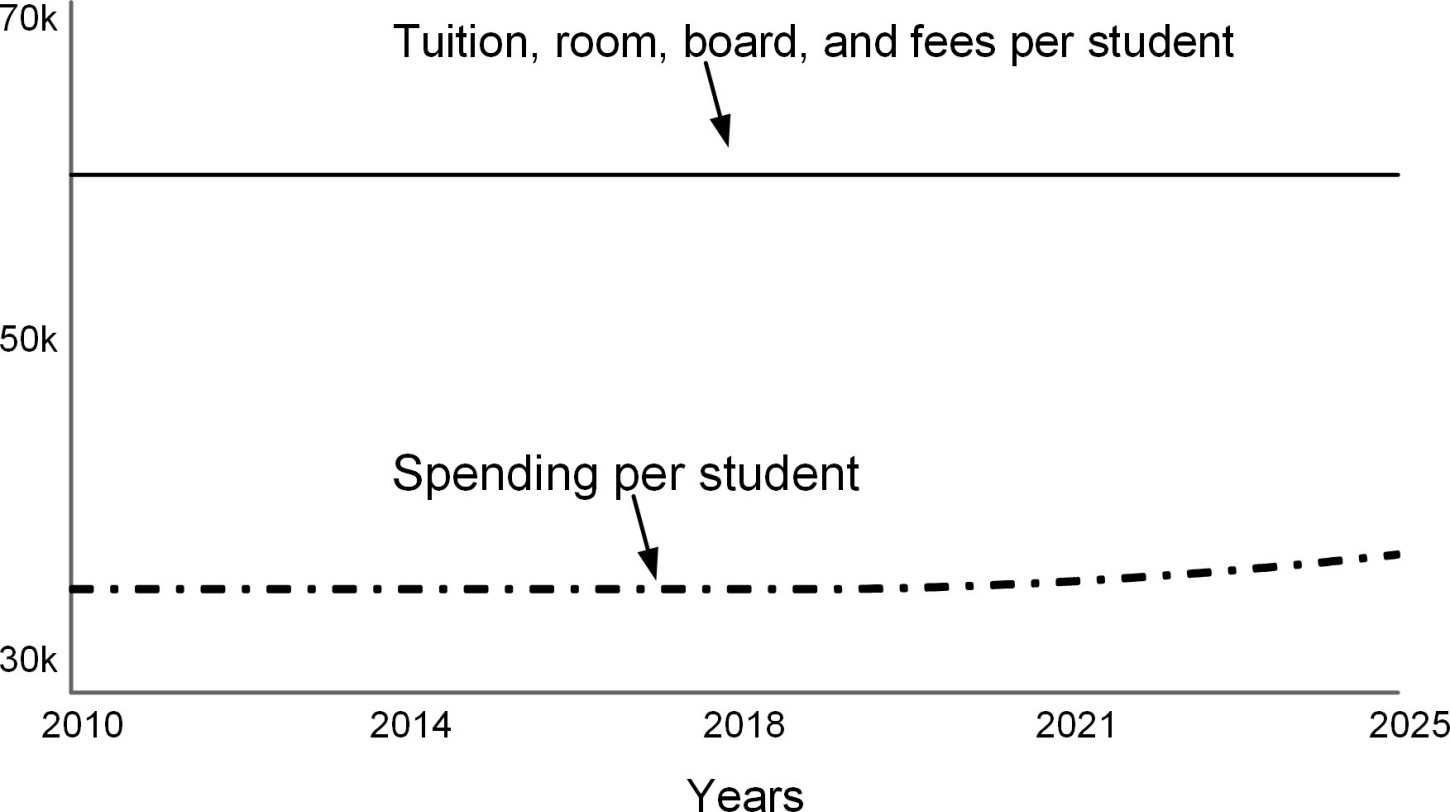
Metrics per student.

The annual spending per student increases (dashed curve in Fig 12) as expenses are distributed over fewer students. Note that this model assumes that tuition, room, board, and fees (solid line in Fig 12) stay constant. At colleges that do not have significant endowments, full-paying students subsidize students who receive scholarships [4]. When spending per student approaches the sticker price, the college’s ability to offer financial aid is reduced.

#### Scenario 2: Reduce faculty (cost strategy)

A college may respond to lower enrollments by reducing the number of faculty to cut operating expenses, as discussed earlier. The following runs simulate the scenario when the college reduces the number of faculty by cutting new searches starting in 2015, the year that student applications begin to decline.

**Fig 13:**
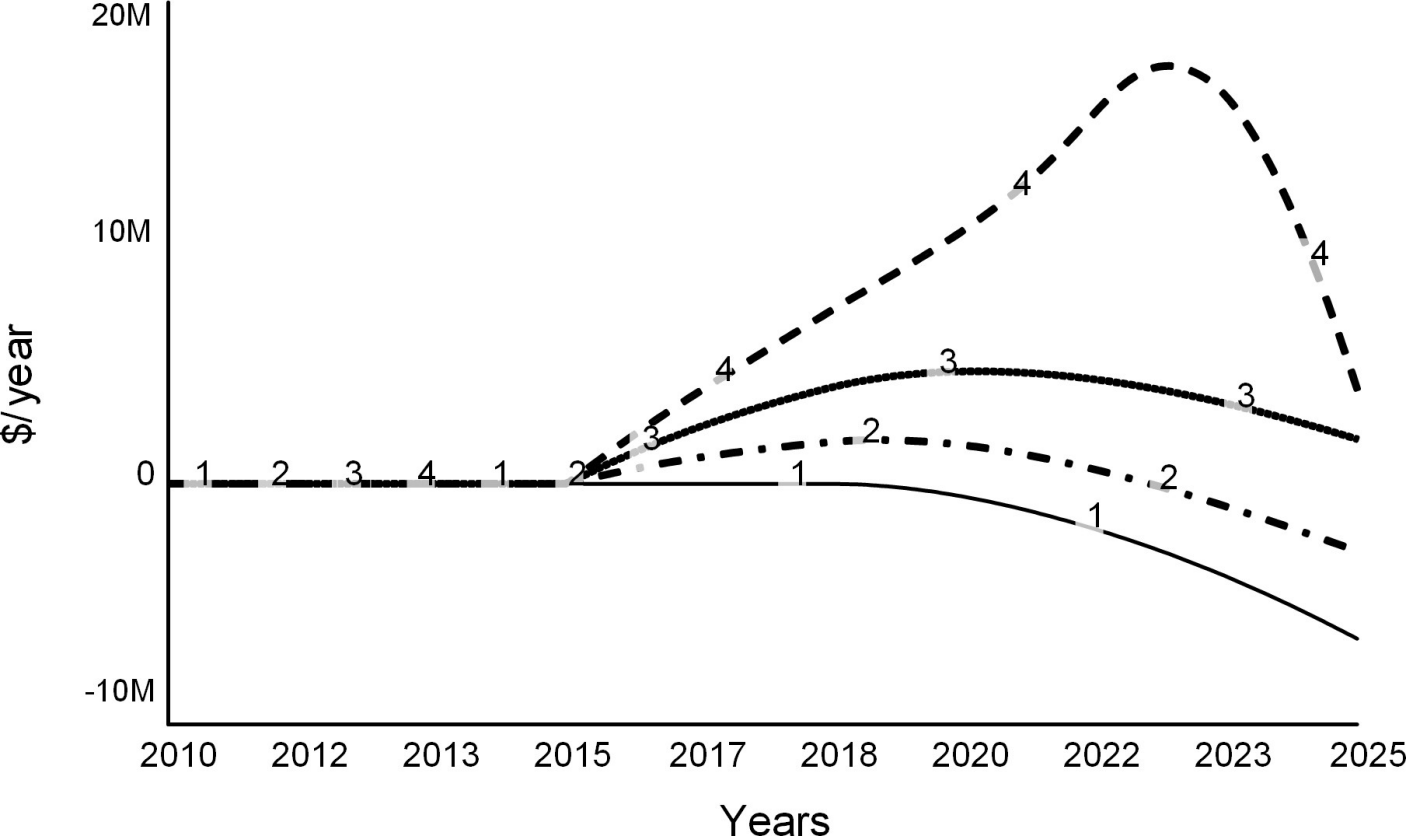
Net revenue at different levels of hiring. Curve 1 is the base run: all searchers are allowed. Curve 2: 75% of searches allowed. Curve 3: 50% of searchers allowed, and Curve 4: 25% of searches allowed.

**Fig 14:**
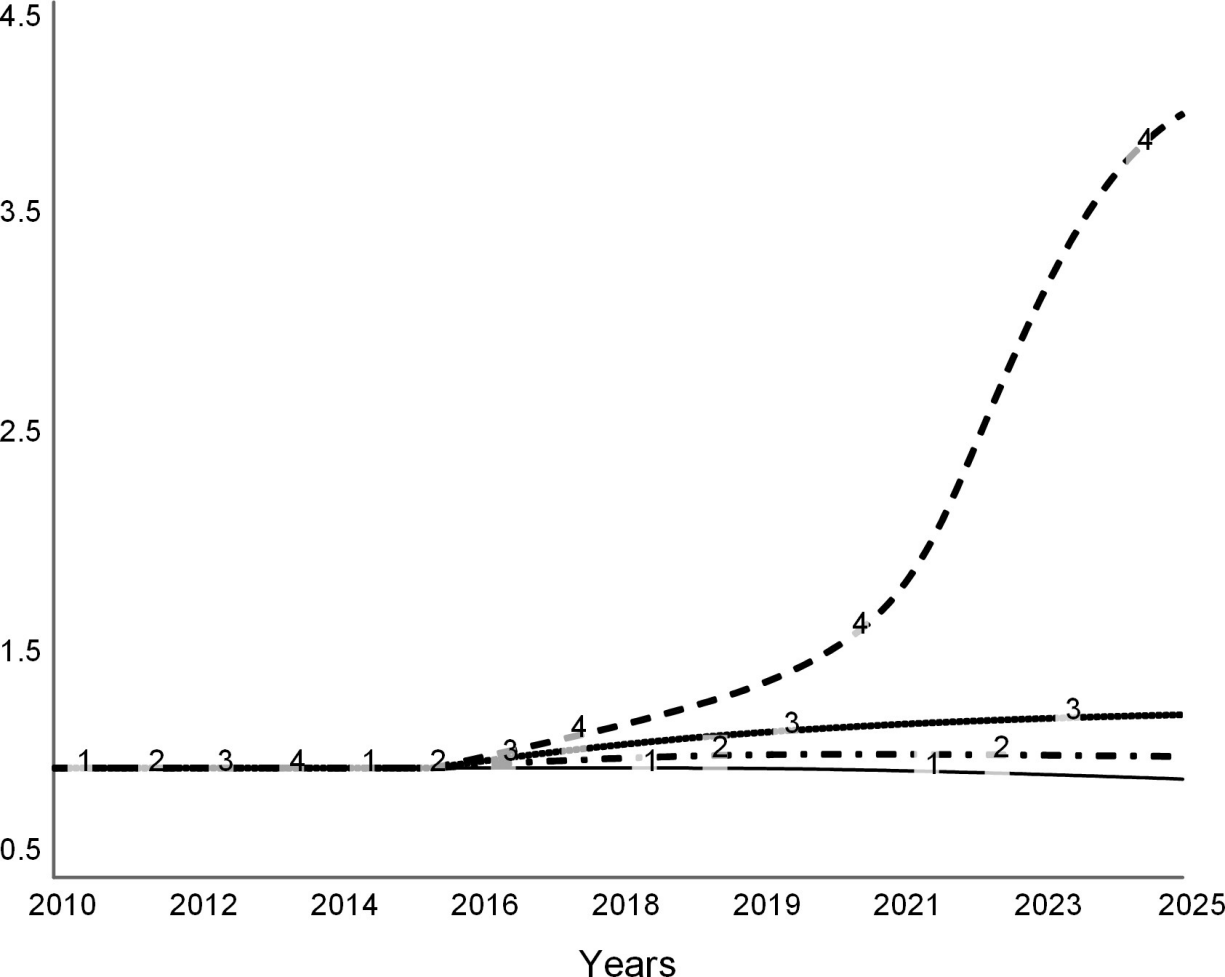
Faculty load increases as the college hires fewer faculty. Curve 1 is the base run: all searchers are allowed. Curve 2: 75% of searches allowed. Curve 3: 50% and Curve 4: 25% of searches allowed.

**Fig 15:**
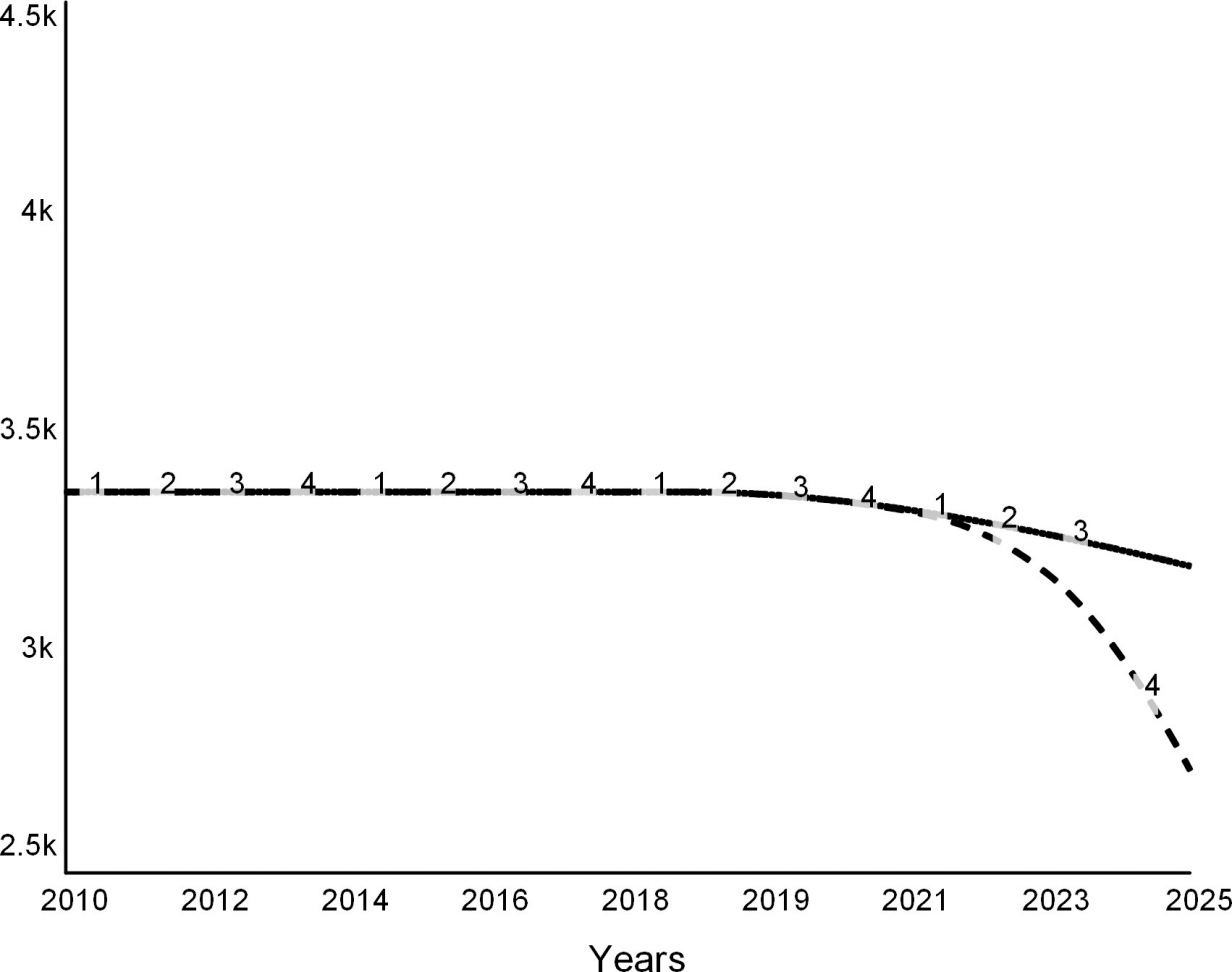
Student enrollments. Curve 1 is the base run: all searchers are allowed. Curve 2: 75% of searches allowed. Curve 3: 50% of searches allowed, and Curve 4: 25% of searches allowed.

Figs 13, 14 and 15 show trajectories for different levels of allowed faculty hiring:

- Curve 1 in all figures is the base run from the “do nothing” scenario, during which all desired searchers are permitted. The run starts in the steady state with zero net revenue (Fig 13). After 2019, the college experiences budget deficits (negative net revenue). It never recovers, as curve 1 stays below zero.
- Curve 2 is for the experiment when 75 percent of all desired searches are allowed. Despite the decline in revenue due to lower enrollments, the expenditure cuts are sufficient for the college to experience a temporary surplus surge until 2023 (Fig 13). After 2023, the college runs an operating deficit. The few remaining faculty experience higher teaching loads (curve 2 in Fig 14). Student enrollments are not significantly different from the base run (Fig 15).
- Curve 3 is a trajectory for the case that allows for 50 percent of searches. Net revenue stays positive through the simulation (Fig 13). At this level of hiring, the faculty teaching load is higher than during the base run (Fig 14), which encourages more faculty attritions. Student enrollments are not significantly different from the base run (Fig 15).
- Curve 4 is for the case when only 25 percent of faculty searches are allowed by the administration. The college experiences a spike in surplus (Fig 13). The faculty teaching load increases dramatically (Fig 14). Student enrollments are the lowest (Fig 15) of all the cases due to the low reputation of the college.

This set of experiments suggests that carefully tuned faculty reductions may offer a short-lived financial reprieve. However, reducing faculty may have long-term adverse effects, and therefore the college may need to find other long-term solutions.

Note that this analysis understates the adverse effects of faculty cuts. The model does not consider several secondary effects of the cost strategy such as fewer courses, a modest selection of majors and minors, scarce academic support, and negative morale on campus and across alumni. Moreover, reporting by the media and public discussions on social media are likely to heighten the harmful effects of faculty cuts.

#### Scenario 3: Invest in facilities (revenue strategy)

Colleges invest in facilities to improve their competitive standing, which they hope will attract more students and improve their revenue [6, 22]. As the president of one troubled liberal arts college stated, the college “…could invest in new facilities to improve its application and retention rates, ultimately reversing the vicious cycle of underwhelming enrollment trends and tuition dependence into a virtuous one of growing demand and a diversified financial portfolio” [7]. The following set of experiments explores this scenario.

**Fig 16:**
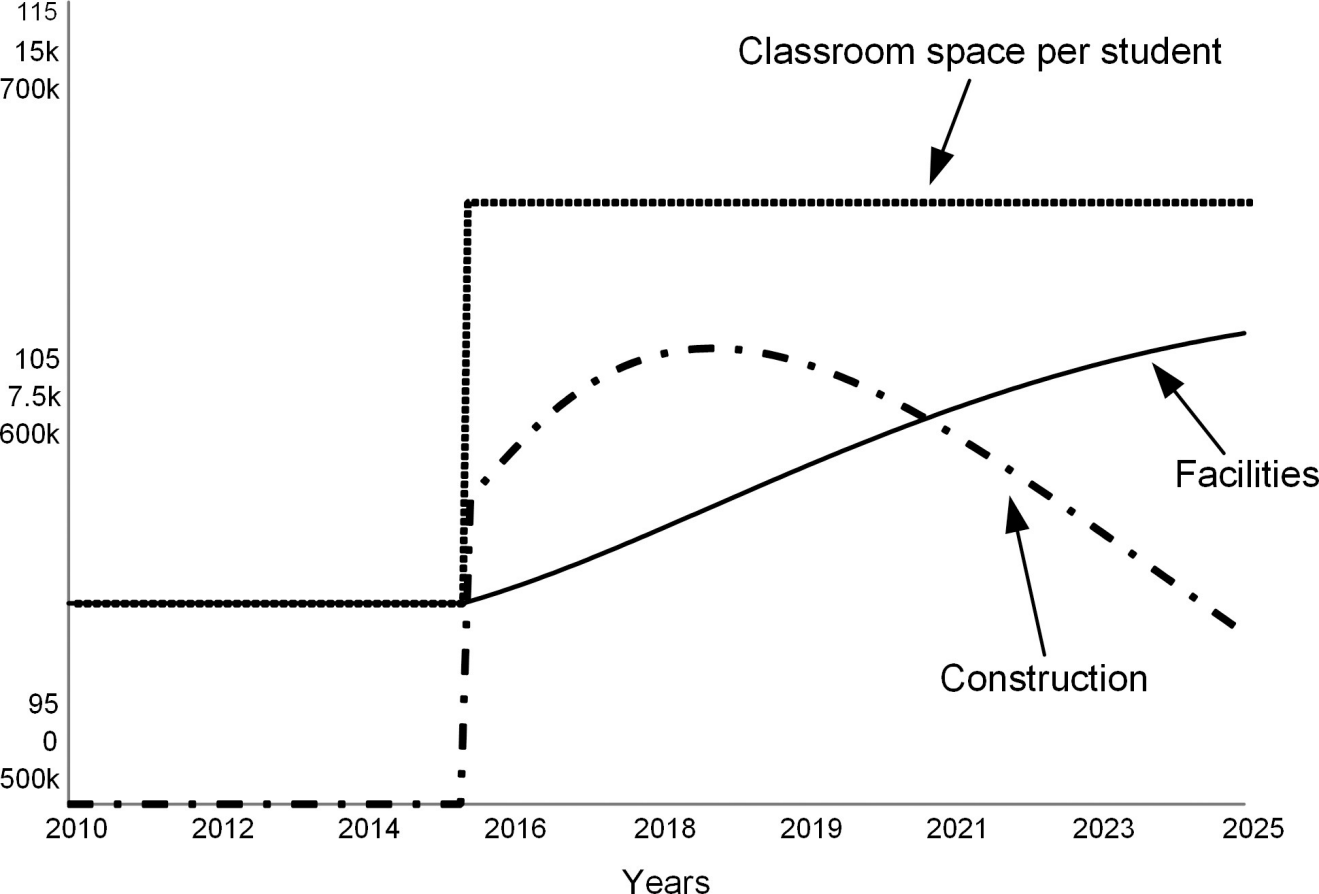
Facilities hike. In 2015, college decides to increase classroom space per student, and therefore starts new construction. Over time, the amount of available facilities increases to satisfy the new standard.

These simulations assume that to increase its competitiveness and improve enrollments, the college leadership commits to expanding the classroom space per student by 10 percent. In the simulation, we model this decision as a step function in 2015 (dotted line in Fig 16). This decision leads to new construction (dashed curve in Fig 16) that increases the stock of campus facilities over time (solid curve in Fig 16). Following the common practice [24], the model assumes that the college borrows funds for new construction.

**Fig 17:**
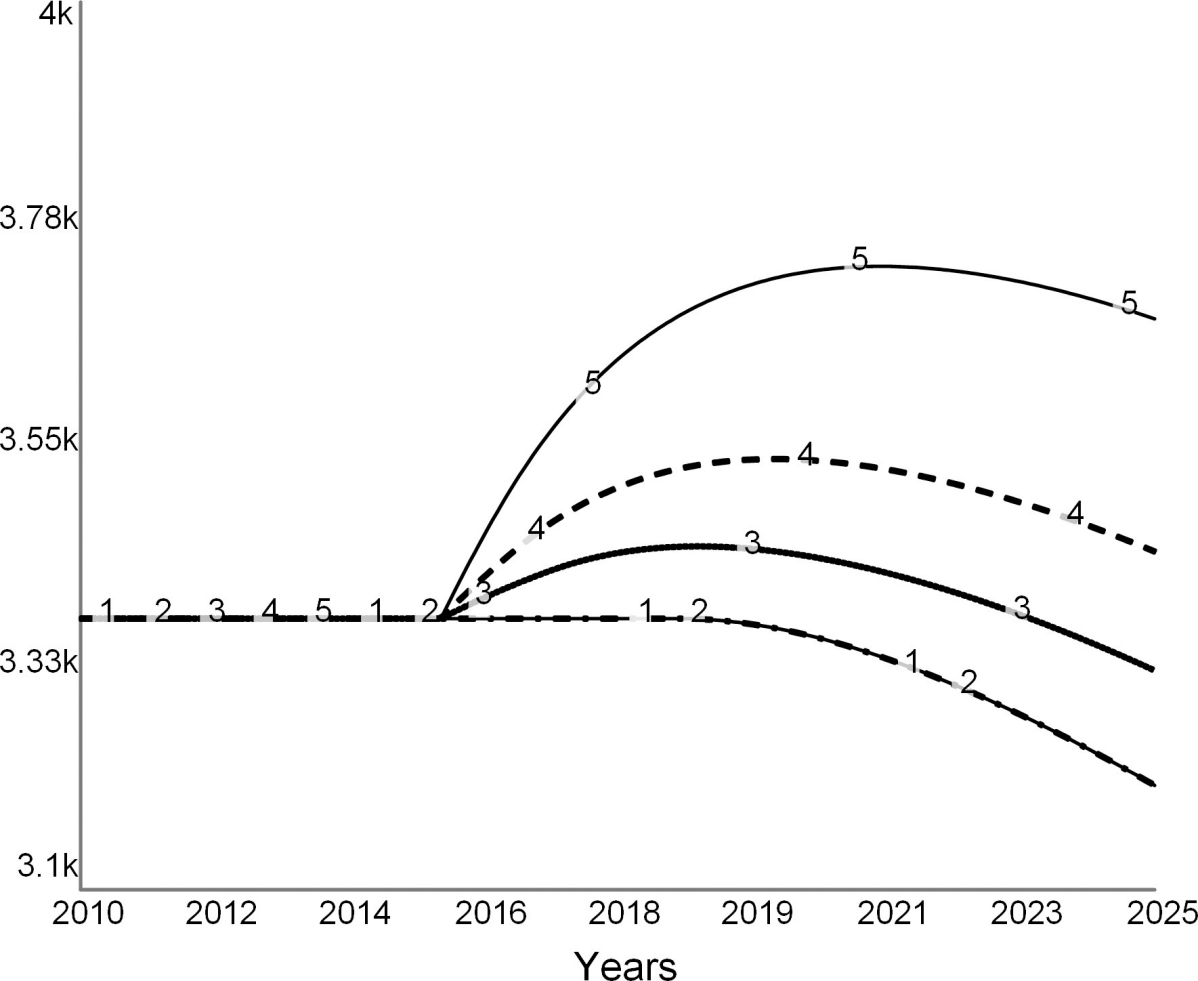
Student enrollments. Spending on facilities might give a short reprieve. Curve 1: base run. Curve 2: 0% increase in enrollments. Curve 3: 5% increase in enrollments. Curve 4: 10% increase in enrollments. Curve 5: 20% increase in enrollments.

**Fig 18:**
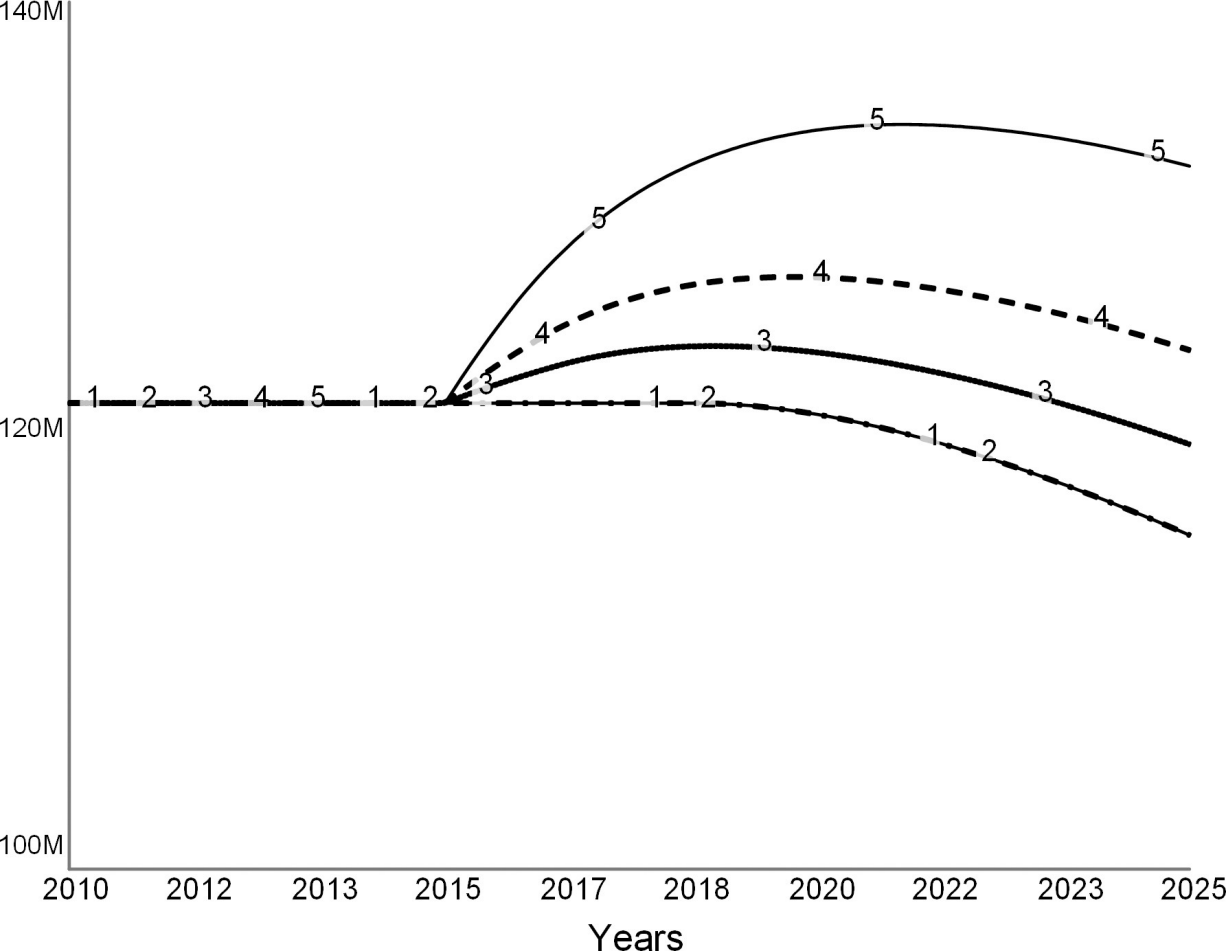
Revenue. Curve 1: base run. Curve 2: 0% increase in enrollments. Curve 3: 5% increase in enrollments. Curve 4: 10% increase in enrollments. Curve 5: 20% increase in enrollments.

**Fig 19:**
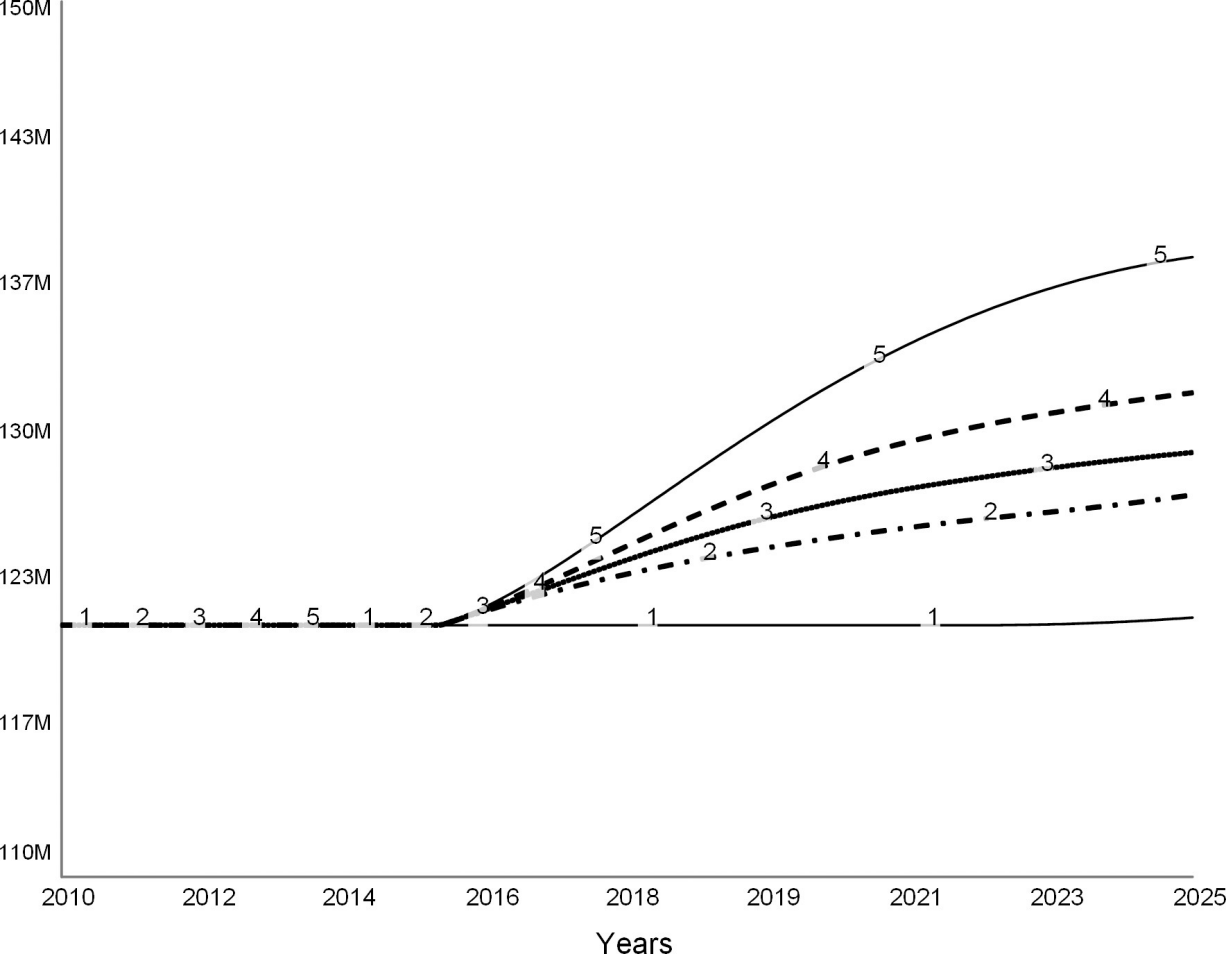
Expenses. Curve 1: base run. Curve 2: 0% increase in enrollments. Curve 3: 5% increase in enrollments. Curve 4: 10% increase in enrollments. Curve 5: 20% increase in enrollments.

**Fig 20:**
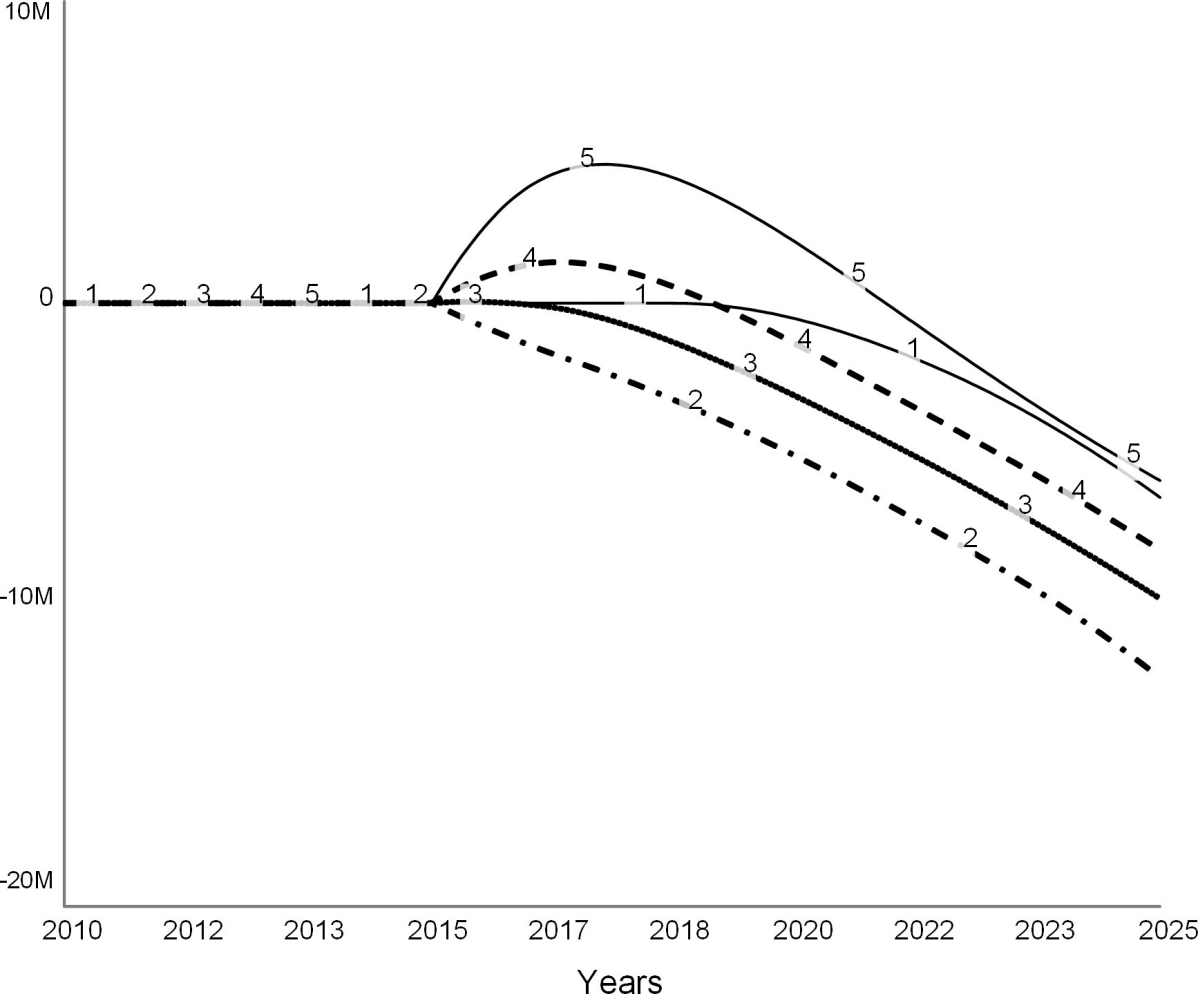
Net revenue. Curve 1: base run. Curve 2: 0% increase in enrollments. Curve 3: 5% increase in enrollments. Curve 4: 10% increase in enrollments. Curve 5: 20% increase in enrollments.

In Figs 17–20, curve 1 is the base run, the “do nothing” scenario. Curve 2 shows a case when, despite better facilities, enrollments do not increase over the base run. Curves 3, 4, and 5 show cases when due to new facilities the incoming classes are 5, 10, and 20 percent larger than in the base case. Curve 5 is the most optimistic case for the college.

Fig 17 depicts changes in student enrollments. Curve 1 of the base run coincides with curve 2, as expected. Curves 3, 4, and 5 show increased enrollments. Fig 18 shows corresponding revenue changes. When there are no changes in enrollments, revenue does not change (curve 2) from the base run (curve 1). When enrollments increase (curves 3, 4, and 5), tuition revenue increases. The college earns the highest revenue when enrollments increase by 20 percent (curve 5).

Fig 19 compares operating expenses for the base run (curve 1) to four additional cases (curves 2–5). The operating expenses increase due to the interest on new debt that financed construction, and the cost of operating the new facilities. In addition, more students imply that the college needs to maintain a larger teaching staff, which also adds to the operating cost as salaries.

Fig 20 shows the net revenue for the five simulations, including the base run (curve 1). In the short term, the college experiences surpluses if enrollments increase by at least 10 percent (curves 4 and 5). However, in the long term, expenses wipe out the new revenue from additional student enrollments.

#### Summary of results

Table 1 summarizes results for the three scenarios discussed above over the short and long term. Enrollments begin to decline in 2015. This is also the year when the model implements policies aimed at mitigating the decline. The next column shows indicator values for 2017, two years into the policies. Ten-year values are in the last column; this is the long term. For the cost strategy, we show the performance when 75 percent of faculty searches were allowed. The revenue strategy shows the case when the incoming class jumps by 20 percent.

**Table 1:** College performance in the short (2 years) and long term (10 years).

		Performance		
Scenario	Indicators	Initial (2015)	Short-term (2017)	Long-term (2025)
**Do nothing**				
	Incoming class	750	750	672
	Students	3,375	3,375	3,206
	Faculty	675	675	675
	Facilities	550,125	550,125	550,125
	Net revenue	0	0	-6,438,471
	Spending per student	36,000	36,000	38,008
	Endowment	50M	50M	41M
	Debt	0	0	6M
**Cost strategy**				
75% of faculty searches allowed	Incoming class	750	750	672
	Students	3,375	3,375	3,206
	Faculty	675	650	609
	Facilities	550,125	550,125	550,125
	Net revenue	0	1,236,066	-2,795,535
	Spending per student	36,000	35,634	36,872
	Endowment	50M	50M	50M
	Debt	0	0	0
**Revenue strategy**				
20% increase in incoming class	Incoming class	750	900	806
	Students	3,375	3,614	3,773
	Faculty	675	688	753
	Facilities	550,125	564,258	627,505
	Net revenue	0	4,859,725	-5,487,452
	Spending per student	36,000	34,655	37,454
	Endowment	50M	50M	50M
	Debt	0	13M	56M

All three scenarios start in the same state in 2015. However, the short-term and long-term outcomes are different for the three strategies. The “do nothing” scenario demonstrates the negative consequences feared by colleges. In the long run, it shows a significantly lower incoming class, lower student enrollments, negative net revenue (i.e. operating deficit), higher expenditure per student, lower endowment, and a substantial debt. In the base case, neither faculty nor facilities change over the ten years. While the college can function for a few more years if it funds operations from the endowment and gifts, the trend is not sustainable in the long run. The two other scenarios are attempts to improve the situation.

In the second scenario, the faculty levels are the lowest of the three scenarios. In the short term, by having fewer faculty, the college improves its financial situation, as there is a surplus, and spending per student is lower. Even after ten years, no withdrawals from the endowment are necessary, and the college does not borrow for operations. The deficit, which is the smallest of the three scenarios, is paid from the cash reserve accumulated over the prior years. The long-term expenditure per student increases as compared to 2015; however, it is the lowest of the three scenarios.

In the third scenario, the college increases facilities per student by 10 percent, which it hopes would improve its competitiveness and lead to more enrollments and revenue. In the short term, the college attracts the incoming class of 900 students, which is the largest class in the three scenarios. To accommodate more students, the college hires more faculty. In the short run, due to the surge in tuition revenue, college experiences a significant surplus. As the college borrows funds for construction, in the long term, the expansion results in a substantial debt. Due to the surplus in preceding years, the college amasses a significant cash reserve that it uses to cover the operational deficit. The college manages to preserve its endowment intact.

## Discussion

We now discuss implications for practice and insights from our results in a form that is most relevant to academic leaders and policy-makers. It is important to note that our simulations do not provide precise forecasts for any given college. However, the model explains the general effects and consequences of the two strategies aimed at mitigating declining college enrollments.

Table 2 highlights the pros and cons of the three scenarios. Simulations suggest that the “do nothing” strategy allows maintaining the status quo in the short term; however, it is not sustainable in the long run. Because the college does not have any surplus, it cannot accumulate cash reserves. Hence, it must draw from the endowment and borrow for operations when it experiences a deficit.

**Table 2:** Scenario highlights with pros (✓) and cons (✘).

Scenario	Short-term (2 years)	Long-term (10 years)
**Do nothing**	✓ Same number of students	✘ Fewer students
	✓ Same faculty	✓ Same faculty
	✓ Same facilities	✓ Same facilities
	✘ No surplus	✘ Highest deficit
	✓ Same spending per student	✘ Highest spending per student
	**✓** Preserved endowment	✘ Lower endowment
	✓ No debt	✘ Operations financed by debt
**Cost strategy**	✓ Same number of students	✘ Fewer students
	✘ Fewer faculty	✘ Fewer faculty
	✓ Same facilities	✓ Same facilities
	✓ Surplus	✘ Smallest deficit
	✓ Lower spending per student	✘ Higher spending per student
	✓ Preserved endowment	✓ Preserved endowment
	✓ No debt	✓ No debt
**Revenue strategy**	✓ More students	✓ More students
	✓ More faculty	✓ More faculty
	✓ More facilities	✓ Significantly more facilities
	✓ Surplus	✘ Deficit
	✓ Lower spending per student	✘ Higher spending per student
	✓ Preserved endowment	✓ Preserved endowment
	✘ Debt	✘ Significant debt

The cost strategy reduces the number of faculty, creates a surplus, and, in the short term, lowers the spending per student. The number of students in the long run is the same, as in the “do nothing” scenario. The college manages to preserve the endowment and accumulates no debt. However, the college still runs operating deficit, even though the deficit is the smallest of the three scenarios.

In the third scenario, the college reverses declining enrollments. It attracts more students, but it incurs debt as the college borrows for construction. Within ten years, the college has more faculty, more students, and more facilities. However, it also runs a deficit, which is not sustainable in the long run, unless tuition increases sufficiently to cover the operating deficit.

*While the cost strategy leads to the least damaging financial situation for the college*, *neither of the strategies are sustainable in the long term because each of them results in an operating deficit*. Moreover, after 10 years, average spending per student is higher under each strategy, which would add to the pressure for the college to increase tuition. While continuous tuition escalation has sustained colleges in the past [4, 22], economic theory [65] suggests that increasing tuition might be a counterproductive approach at the time when demand for college is shrinking. Possible better solutions include encouraging higher college attendance rates [2], reengineering universities as data-driven institutions [12, 66], encouraging campus innovation for additional revenue streams [5], or completely redesigning the higher education business model [67].

## Conclusion

There is an approaching “storm” in the U.S. undergraduate student market. As the college-age population declines, the tuition-dependent colleges need to adapt to the demographic change. Motivated by this problem, this article conducts a *model-driven analysis* of three plausible scenarios. It draws on systems theory to conceptualize a *college as a complex service system* and to develop a system dynamics computational model that captures core causal interrelationships and multiple feedback effects between faculty, facilities, tuition revenue, financials, reputation, and outcomes. The resulting *college model* allows performing simulations that test the short-term and long-term financial viability of a college. The analysis suggests that common solutions such as cutting cost by reducing faculty or improving campus facilities to attract students and increase revenue may improve the college’s short-term financial position. However, these strategies are insufficient to ensure the long-term viability of the college without the continuous tuition hikes.

The main contribution of this article is a computational model that adds to our understanding of higher education economics, management and strategy. It can be used for *model-driven academic management* that supplements traditional planning at colleges. The analysis of this feedback-rich model provides insights that can inform college leaders and policy-makers. The computational model and model-driven analysis can be used together with such strategy tools as SWOT (Strengths, Weaknesses, Opportunities and Threats) [3, 68] and PESTLE (Political, Economic, Social, Technical, Legal and Environmental) [69] that examine the impact of external environmental factors on colleges.

The computational model can also provide value as part of an interactive learning environment that can help with dynamic decision making [70]. System-based learning and planning environments can improve performance and decision-making on several scales including decision heuristics, structural knowledge, decision time and decision strategy [71, 72], especially when combined with prior exploration [73] and debriefing [74–77].

Limitations of this model can provide fruitful topics for future research. This version of the model does not analyze the effects of interest rates, market returns, marketing, yield determination, and discount rates. These variables might be critical for marginally viable colleges. Therefore, we plan to consider these variables in the future extensions of the model that would allow new strategies in addition to the ones studied in this article. The student sector can be expanded to include academic advising and co-curricular activities that influence the retention rates. We could also review combined strategies. Future research could also consider how proliferation and improvement of digital technologies [78] may alter demand for on-campus education.

## Appendix A: Alternative application decline rates

To examine how the results vary if the decline rate is lower than 15 percent, we have performed additional simulations. Table 3 provides performance outcomes for a college when applications decline by five percent and Table 4 shows results when applications drop by 10 percent. The simulations show that the college can easily weather a five percent decline. When applications drop by five percent (Table 3), the college can still maintain its status quo in the short and long term without any strategic adjustments. This is because the college still receives enough applications to recruit a sufficiently large incoming class. Considering that the student population stays constant, the cost strategy leads to a smaller faculty size, which implies a greater than normal faculty workload–not a desirable outcome. Because the college earns a surplus, there is no need to borrow for operations.

**Table 3:** College performance when application decline by five percent.

		Performance		
Scenario	Indicators	Initial (2015)	Short-term (2017)	Long-term (2025)
**Do nothing**				
	Incoming class	750	750	750
	Students	3,375	3,375	3,375
	Faculty	675	675	675
	Facilities	550,125	550,125	550,125
	Net revenue	0	0	0
	Spending per student	36,000	36,000	36,000
	Endowment	50M	50M	50M
	Debt	0	0	0
**Cost strategy**				
75% of faculty searches allowed	Incoming class	750	750	750
	Students	3,375	3,375	3,375
	Faculty	675	650	624
	Facilities	550,125	550,125	550,125
	Net revenue	0	1,236,066	2,551,047
	Spending per student	36,000	35,634	35,244
	Endowment	50M	50M	50M
	Debt	0	0	0
**Revenue strategy**				
20% increase in incoming class	Incoming class	750	900	900
	Students	3,375	3,614	3,976
	Faculty	675	688	775
	Facilities	550,125	564,258	636,269
	Net revenue	0	4,859,725	-1,121,955
	Spending per student	36,000	34,655	36,282
	Endowment	50M	50M	50M
	Debt	0	13M	64M

**Table 4:** College performance when application decline by 10 percent.

		Performance		
Scenario	Indicators	Initial (2015)	Short-term (2017)	Long-term (2025)
**Do nothing**				
	Incoming class	750	750	711
	Students	3,375	3,375	3,305
	Faculty	675	675	675
	Facilities	550,125	550,125	550,125
	Net revenue	0	0	-2,508,342
	Spending per student	36,000	36,000	36,759
	Endowment	50M	50M	46M
	Debt	0	0	27,795
**Cost strategy**				
75% of faculty searches allowed	Incoming class	750	750	711
	Students	3,375	3,375	3,305
	Faculty	675	650	619
	Facilities	550,125	550,125	550,125
	Net revenue	0	1,236,066	288,146
	Spending per student	36,000	35,634	35,913
	Endowment	50M	50M	50M
	Debt	0	0	0
**Revenue strategy**				
20% increase in incoming class	Incoming class	750	900	853
	Students	3,375	3,614	3,892
	Faculty	675	688	767
	Facilities	550,125	564,258	633,540
	Net revenue	0	4,859,725	-3,185,005
	Spending per student	36,000	34,655	36,818
	Endowment	50M	50M	50M
	Debt	0	13M	62M

Under the revenue strategy, the institution grows–there are more students, faculty, and facilities. In the short term, the college earns a significant operating surplus, which, however, comes at the cost of significant debt due to additional construction. In the long run, continued construction adds to the debt, and, without a tuition hike, the college would face operating deficit (negative net revenue). The endowment can be preserved in the short and long term. *To sum up*, *if applications decline by five percent*, *the “do nothing” strategy is sustainable*.

A 10 percent decline in applications worsens the situation so that a “do nothing” strategy is no longer acceptable (Table 4). In the long term, the college experiences operating deficit that it covers by drawing from the endowment and borrowing. The cost strategy allows the college to have a positive new revenue (a surplus) for the next 10 years, which eliminates the need to draw from the endowment and borrow for operations. The revenue strategy always leads to the largest student body and the most faculty among the three scenarios. The college earns a significant surplus in the short run, but the surplus turns into a large operating deficit by 2025. The college accumulates a significant debt. *In summary*, *if faced with a 10 percent drop in applications*, *the “do nothing” strategy is not sustainable and the college would be well advised to pursue the cost strategy*.

## Appendix B: Model equations

**Fig 21:**
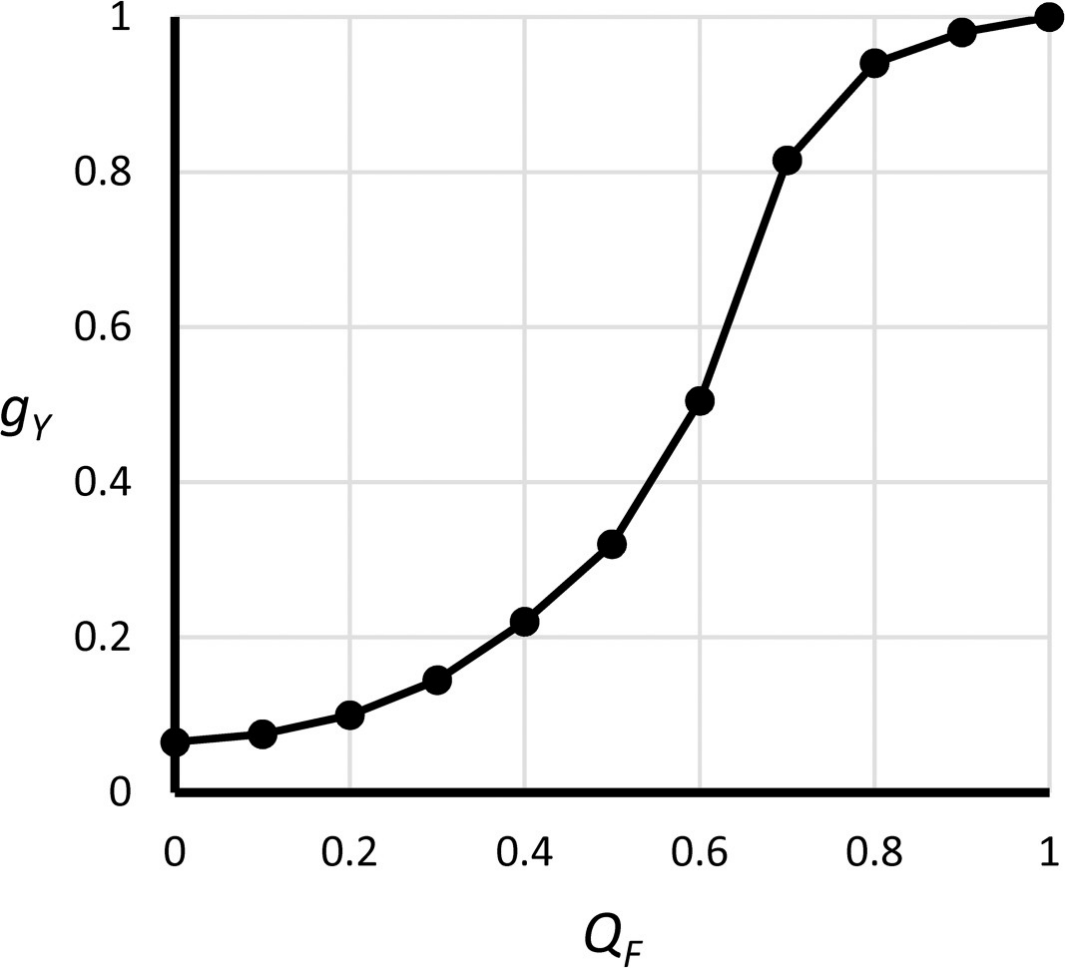
Graphical function *g*_*Y*_(*Q*_*F*_).

**Fig 22:**
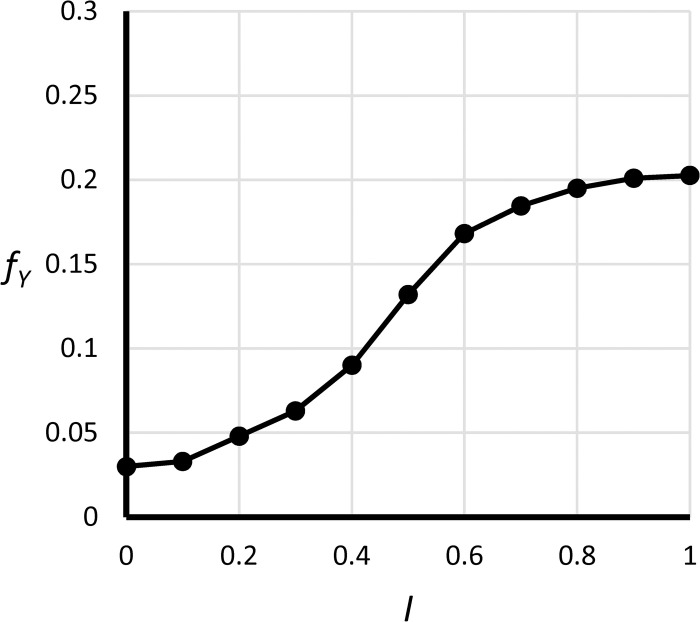
Graphical function *f*_*y*_(*I*).

**Fig 23:**
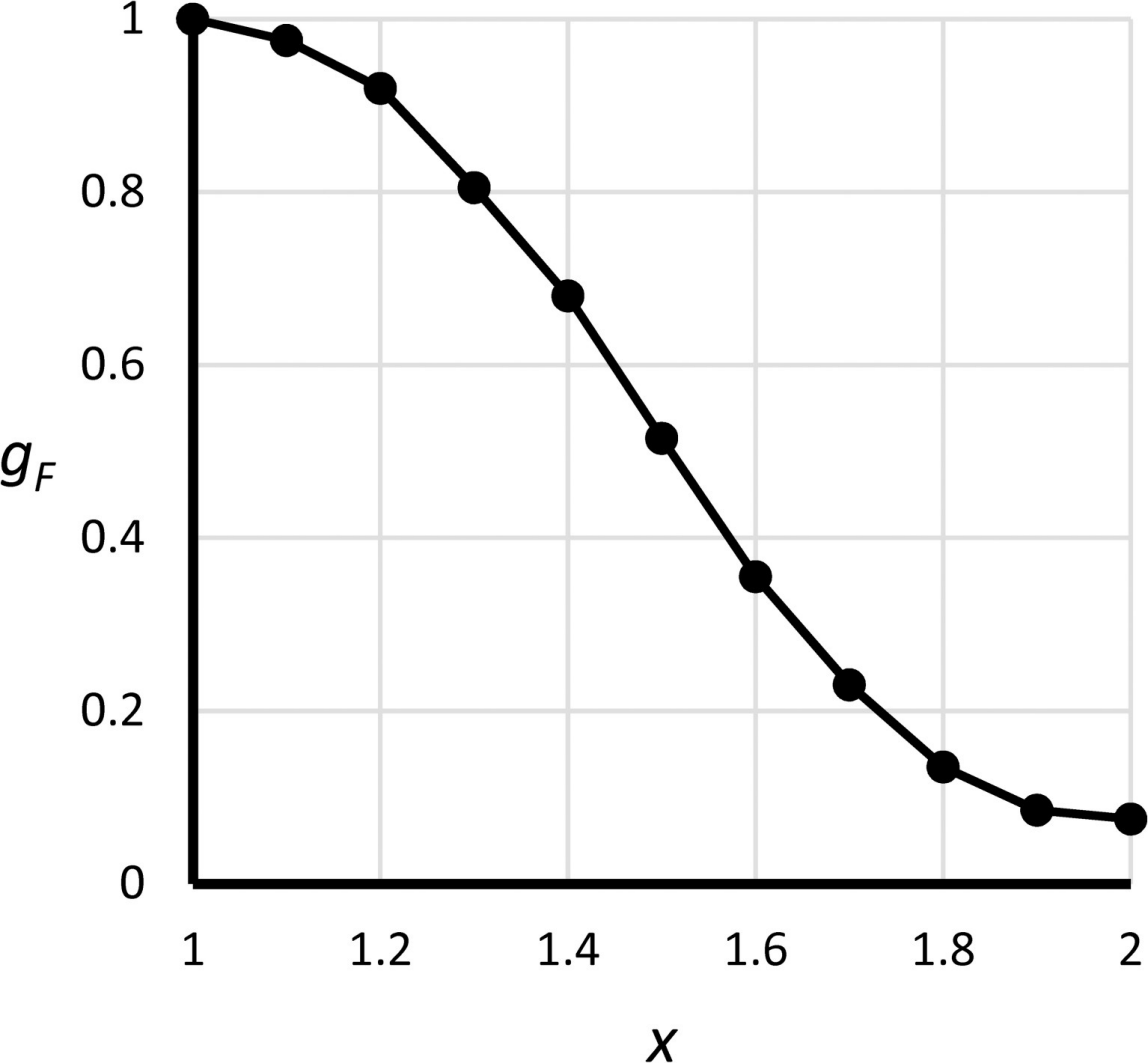
Graphical function *g*_*F*_(*x*). Here *x* = (*l*_*F*_+0.5max (1,*γ*_*B*_))/1.5 is a composite index consisting of faculty academic load index *l*_*F*_ and facilities loading *γ*_*B*_.

This section shows equations for the computational model of a typical tuition-dependent college. This model augments and modifies an earlier model developed by Zaini et al. [62]. Mathematically, the model is a system of non-linear integral equations. It was implemented and simulated in the system dynamics modeling software called *Stella Architect* available from *isee systems*. In the following tables, variables are arranged by sectors–Students, Faculty, Facilities, and Financials. The tables show parameter values and initial conditions for stocks in the steady state. Figs 21–23 show graphical functions used in the model. The model is unit-consistent.

**Table:** 

**Students**			
*Variable*	*Formula*	*Unit*	*Notes*
Applicants	*A* = 6500	students/year	Selective colleges have significantly more applications than the college can admit.
Admission rate	*λ*_*Y*_ = 0.6	dimensionless	Selective colleges have lower admission rates.
Student satisfaction	*Q*_*Y*_ = *g*_*Y*_(*Q*_*F*_), $g_{Y}^{\prime }(Q_{F})>0$	dimensionless	Student satisfaction is a function of faculty academic experience. College reputation depends on student satisfaction. See Fig 21 for definition of *g*_*Y*_(∙).
College reputation	*I* = {*Q*_*Y*_}	dimensionless	College reputation is an exponential smoothing of student satisfaction.
Yield rate	*y* = *f*_*y*_(*I*), $f_{y}^{\prime }(I)>0$	dimensionless	A fraction of admitted students who enroll. See Fig 22 for definition of *f*_*y*_(∙).
Yield	*i*_*Y*_ = *yλ*_*Y*_*A*	students/year	Yield is the incoming class.
Graduates per year	*o*_*Y*_ = *Y*/*τ*_*Y*_	students/year	*τ*_*Y*_ is the average stay in college
Students	*Y* = ∫(*i*_*Y*_−*o*_*Y*_)*dt*	students	Incoming classes add to the total enrollment and then students graduate. The initial value is *Y*_0_ = 3375 students.
**Faculty**			
*Variable*	*Formula*	*Unit*	*Notes*
Average generated load per student	*l*_*Y*_ = 100	credits	A measure of how many courses a student takes per year
Student to faculty ratio	*q*_*YF*_ = *Y*/*F*	students/faculty	
Standard student to faculty ratio	${\bar{q}}_{YF}=5$	students/faculty	
Average faculty academic load	*L*_*Y*_ = *q*_*YF*_*l*_*Y*_	credits/faculty	Average teaching load
Standard faculty load	${\bar{L}}_{Y}={\bar{q}}_{YF}l_{Y}$	credits/faculty	
Faculty academic load index	$l_{F}=L_{Y}/{\bar{L}}_{Y}$	dimensionless	
Faculty academic experience	*Q*_*F*_ = *g*_*F*_(*l*_*F*_,*Y*_*B*_)	dimensionless	See Fig 23 for definition of *g*_*F*_(∙).
Average time at college	${\bar{T}}_{F}$ = 10	years	Average duration of faculty employment at a college
Time to decide to leave	*τ*_*F*_ = 2	years	Even unhappy faculty don’t leave immediately
Time to hire faculty	*τ*_*h*_ = 2	years	
Faculty shortage	*s*_*F*_ = max(0,*l*_*F*_−1)	dimensionless	A measure of faculty shortage due to the academic load. *s*_*F*_>0
Standard hiring rate	$\eta =\tau_{F}/{\bar{T}}_{F}+s_{F}$	dimensionless	Standard hiring rate equals the hiring to replace standard attrition $\tau_{F}/{\bar{T}}_{F}$ plus the hiring to address the faculty shortage measured by *s*_*F*_
Allowed faculty searches	*λ*_*F*_ = 1	dimensionless	Percent of searches allowed by administration. Ideally, all searchers are allowed, i.e. *λ*_*F*_ = 1. However, only a fraction of needed searches may be allowed, i.e. *λ*_*F*_∈[0,1]
Hiring of new faculty	*h* = *λ*_*F*_*η*_*F*_/*τ*_*h*_	faculty/year	
Attrition rate	$\phi =\tau_{F}/{\bar{T}}_{F}+(1-Q_{F})$	dimensionless	Faculty attrition rate equals standard attrition $\tau_{F}/{\bar{T}}_{F}$ plus attrition 1−*Q*_*F*_ due to academic experience
Faculty attrition	*o*_*F*_ = *ϕF*/*τ*_*F*_	faculty/year	
Faculty	*F* = ∫(*h*−*o*_*F*_)*dt*	faculty	Initial value is $F_{0}=Y_{0}/{\bar{q}}_{YF}$
**Facilities**			
*Variable*	*Formula*	*Unit*	*Notes*
Space per student	*b*_*Y*_ = 100	ft^2^/student	
Classroom and lab space needed	${\bar{B}}_{Y}=b_{Y}Y$	ft^2^	
Space per faculty member	*b*_*F*_ = 315	ft^2^/faculty	
Faculty office space needed	${\bar{B}}_{F}=b_{F}F$	ft^2^	
Space needed	$\bar{B}={\bar{B}}_{Y}+{\bar{B}}_{F}$	ft^2^	Demand for facilities
Facilities loading	$\gamma_{B}=\bar{B}/B$	dimensionless	A measure of facilities utilization
Percent of approved projects	*λ*_*B*_ = 50	percent	We assume that not all construction projects are approved
Approved facilities construction	*b*_*B*_ = *λ*_*B*_(*γ*_*B*_−1)*B*	ft^2^	New facilities that will be built
Construction time	*τ*_*B*_ = 3	years	
Facilities	*B* = ∫(*b*_*B*_/*τ*_*B*_)*dt*	ft^2^	Supply of facilities. We assume there is no depreciation of facilities. Initial value is *B*_0_ = *b*_*Y*_*Y*_0_+*b*_*F*_*F*_0_
**Financials**			
*Variable*	*Formula*	*Unit*	*Notes*
Discount rate	*δ*_*R*_ = 0.4	dimensionless	Fraction of tuition, room, board and fees distributed as financial aid
Sticker price	*P* = 60,000	$/student/year	Tuition, room, board and fees per student
Revenue	*R* = (1−*δ*_*R*_)*PY*+*g*_*u*_	$/year	Revenue from tuition, room, board and fees and unrestricted gifts less financial aid
Expenses	*C* = *C*_*V*_+*C*_*F*_	$/year	All operating costs combined, which includes variable (*C*_*V*_) and fixed costs (*C*_*F*_).
Net revenue	*S* = *R*−*C*	$/year	When positive (negative), referred to as surplus (deficit)
Cash available for operations	*m*_*C*_ = *M*/*τ*_*F*_	$/year	Cash available during the fiscal year *τ*_*F*_ (duration *τ*_*F*_ = 1 year)
Accepted draw rate	*a*_*E*_ = 0.05	dimensionless	The college can withdraw no more than five percent of its endowment per year
Available endowment draw	*m*_*E*_ = *a*_*E*_*E*	$/year	Money that can be taken from the endowment fund for operations
Average faculty salary	*ω* = 50,000	$/faculty/year	
Salaries	*W* = *ωF*	$/year	Salaries of all faculty, *F*
Endowment draw	*o*_*E*_	$/year	Money taken from the endowment fund for operations
Unrestricted gifts	*g*_*u*_ = 0	$/year	These gifts could be used for operations. We assume no gifts.
Restricted gifts	*g*_*r*_ = 0	$/year	Added to the endowment. We assume no gifts.
Cash	*M* = ∫(*S*−*o*_*m*_)*dt*	$	Cash reserve increases when there is a surplus, S, and decreases when cash, *o*_*m*_, is used to pay for operations. *M*_0_ = $0
Debt	*D* = ∫(*d*_*f*_+*m*_*D*_−*p*_*D*_)*dt*	$	Initial value is *D*_0_ = $0. Cost of new facilities is *d*_*f*_, *m*_*D*_ is what college borrows for operations and *p*_*D*_ are annual debt payments.
Endowment	*E* = ∫(*g*_*r*_−*o*_*E*_)*dt*	$	Restricted gifts add to the endowment and endowment draw reduces it. Initial value is *E*_0_ = $50*e*6.
